# Multi‐omics molecular phenotyping reveals the potential mechanisms of chemotherapy response and resistance in small cell lung cancer

**DOI:** 10.1002/ctm2.1728

**Published:** 2024-06-22

**Authors:** Ying Cheng, Jie Hu, Xuan Gao, Bingfa Yan, Zelong Xu, Ying Liu, Jing Zhu, Zhentian Liu, Ying Wang, Junfeng Wang, Ying Xin, Ke Zheng, Yawen Yang, Xuefeng Xia, Xin Yi, Kai Niu, Changliang Yang, Hongxia Cui, Yanrong Wang, Guang Yang, Jie Hao, Peidong Li, Liang Zhang, Zili Li, Hongyu Wang, Yanli Sun, Shubo Zuo, Tianying Du, Jinhua Xu, Gan Zhang, Fei Chen, Ning Ding

**Affiliations:** ^1^ Department of Medical Oncology Jilin Cancer Hospital Changchun China; ^2^ Department of Pulmonary Medicine Zhongshan Hospital Fudan University Shanghai China; ^3^ Department of Pulmonary Medicine Shanghai Geriatric Center Shanghai China; ^4^ Shanghai Respiratory Research Institute Shanghai China; ^5^ State Key Laboratory of Microbial Resources Institute of Microbiology Chinese Academy of Sciences Beijing China; ^6^ GenePlus‐Shenzhen Clinical Laboratory Shenzhen China; ^7^ Geneplus‐Beijing Beijing China; ^8^ Department of Medical Thoracic Oncology Jilin Cancer Hospital Changchun China

Dear Editor,

Small cell lung cancer (SCLC) is a low‐survival malignant lung cancer with mainly extensive stage (ES).^1^, ^2^ A major challenge in treating SCLC is chemotherapy resistance.^3^ However, studies on disease evolution and molecular mechanisms of resistance during chemotherapy are insufficient. Here, we conducted a multicentre, observational study to profile the multi‐omics characteristics of tumour tissue, circulating tumour cell (CTC) and circulating tumour DNA (ctDNA) in Chinese ES‐SCLC patients.

This study enrolled 54 patients, including naïve cohort and relapsed cohort (Figure S1 and Table S1). Except for one patient who had distant metastasis in relapsed cohort, all the patients had ES‐SCLC. According to the different stratification parameters, patients were divided by two manners. One manner was chemo‐resistant vs chemo‐sensitive, according to whether the time from the end of first‐line therapy to disease progression exceeded 90 days (chemotherapy‐free interval); other manner was responders vs non‐responders, according to Response Evaluation Criteria in Solid Tumours (version 1.1), and the patient whose lesions shrank over 30% was defined as responder. The median overall survival (OS) and progression‐free survival (PFS) for all patients were 9.6 m (95% confidence interval [CI]: 7.5‒12.2 m) and 4.5 m (95% CI: 3.4‒5.7 m), respectively. No clinical parameters had a significant effect on prognosis (Table S2). Chemo‐sensitive/response patients had longer PFS (Figure S2), which suggested that different biological contexts may exist.

The detection of ctDNA mutations was highly consistent with tumour results and the tumour mutation burden (TMB) was highly correlated (Figure S3A‒D and Tables S3 and S4), which indicated that ctDNA mutations could be used to monitor mutational changes during treatment with high confidence. As expected, *TP53* and *RB1* mutations were detected in most patients' baseline ctDNA (Figure S3A). Some frequently deleted genomic regions in tumours and more in CTCs were found (Figure S3E,F), which may indicate the evolution of genomic heterogeneity among diverse clones and the initial development of drug resistance. The tumour showed a high proportion of C > A transitions (Figure S3G).

In both non‐responders and chemo‐resistant in baseline ctDNA, only the *KDR* gene (vascular endothelial growth factor receptor [VEGFR]) had a significantly higher mutation frequency (Figure 1A,C). The baseline ctDNA of chemo‐resistant showed more significant deletion frequency, but only *SORCS1* had a significant deletion frequency in baseline tumours (Figure S4). The TMB of baseline ctDNA in non‐responders and microsatellite instability (MSI) score of baseline tumours in chemo‐resistant were significantly higher (Figures 1B,D and S7B). However, other genomic indexes in tumour had no significant differences (Figures S5‒S7). Three pathways were highly enriched and one pathway was lower in non‐responders (Figure S8).

**FIGURE 1:**
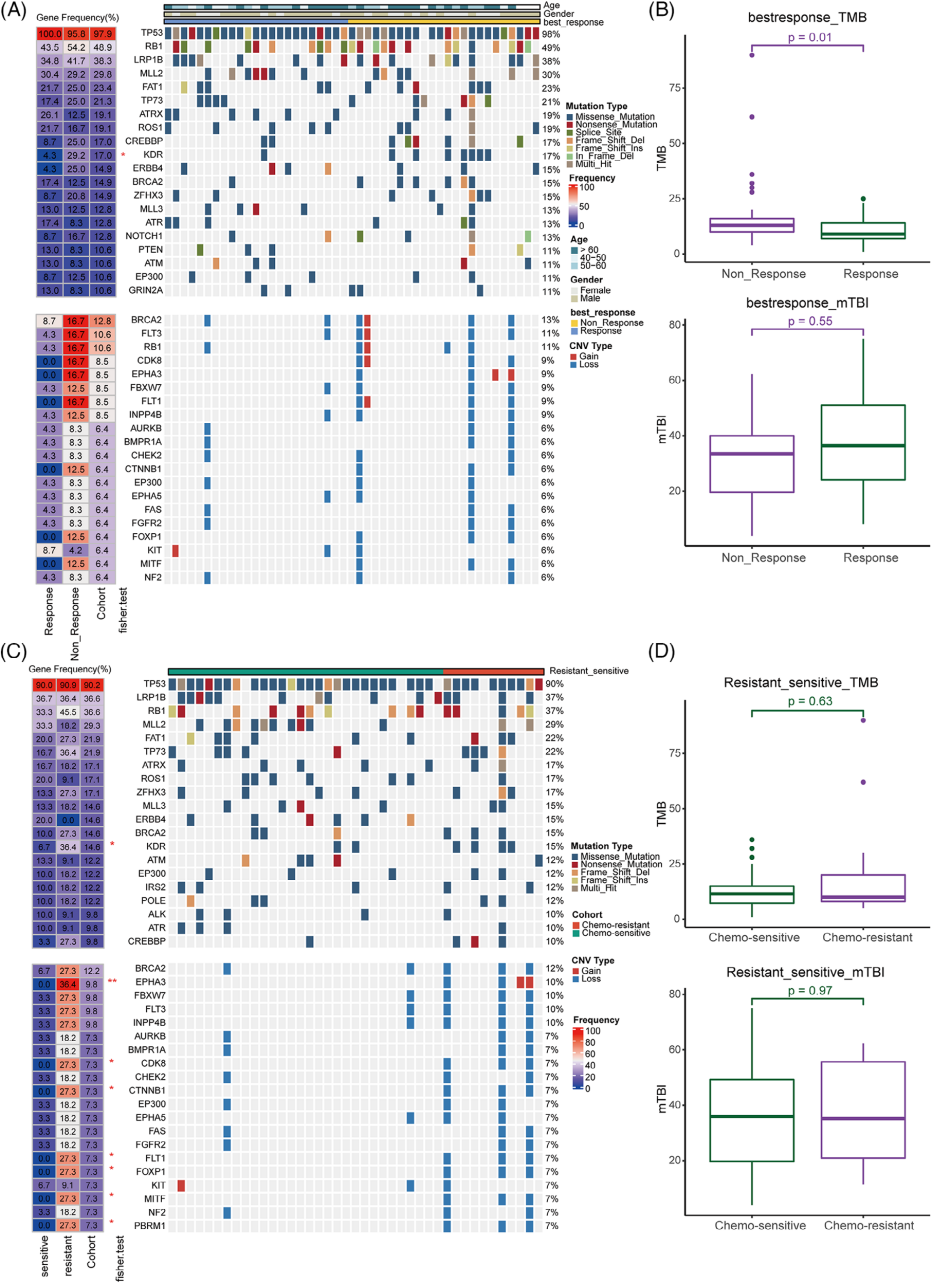
Comparison of somatic mutations and copy number alterations in the response (A) and chemotherapy sensitivity (C) of circulating tumour DNA (ctDNA) samples treated with chemotherapy. The genes with a mutation frequency greater than 10% are shown in heatmap. Each column represents a single sample, and the upper annotation shows the clinical features. The numbers next to the heatmap represent mutation frequency. The significance of the Fisher's exact test results for mutation frequency and corresponding clinical outcome is marked with an asterisk next to the total cohort mutation frequency. Boxplots showing that the tumour mutation burden (TMB) and molecular tumour burden index (mTBI) correlate with responses (B) and chemotherapy sensitivity (D).

Tumour samples clustered into high and low levels of immune infiltrate by RNA‐sequencing (Figure 2A,B). Although immune infiltration had no significant difference (Figure 2C,D), some immune populations in non‐responders/chemo‐resistant were significantly higher (Figures 2E, S9 and S10). The *KRAS* signalling pathways were enriched in non‐responders in tumour (Figures 2F and S11), which were reported to affect the presence and suppressive function of tumouricidal cells.^4^ Conversely, several pathways related to proliferation and immunity were significantly up‐regulated in responders/chemo‐sensitive in both baseline tumours and CTCs (Figures 2F‒I, S11 and S12), suggesting that a more vital ability of differentiation and immunogenicity may occur in chemotherapy‐sensitive tumour. The cell death pathway related to pyroptosis was different in baseline CTCs, and there was a significantly higher score of alkaliptosis in relapse nodes (Figure 2J,K).

**FIGURE 2:**
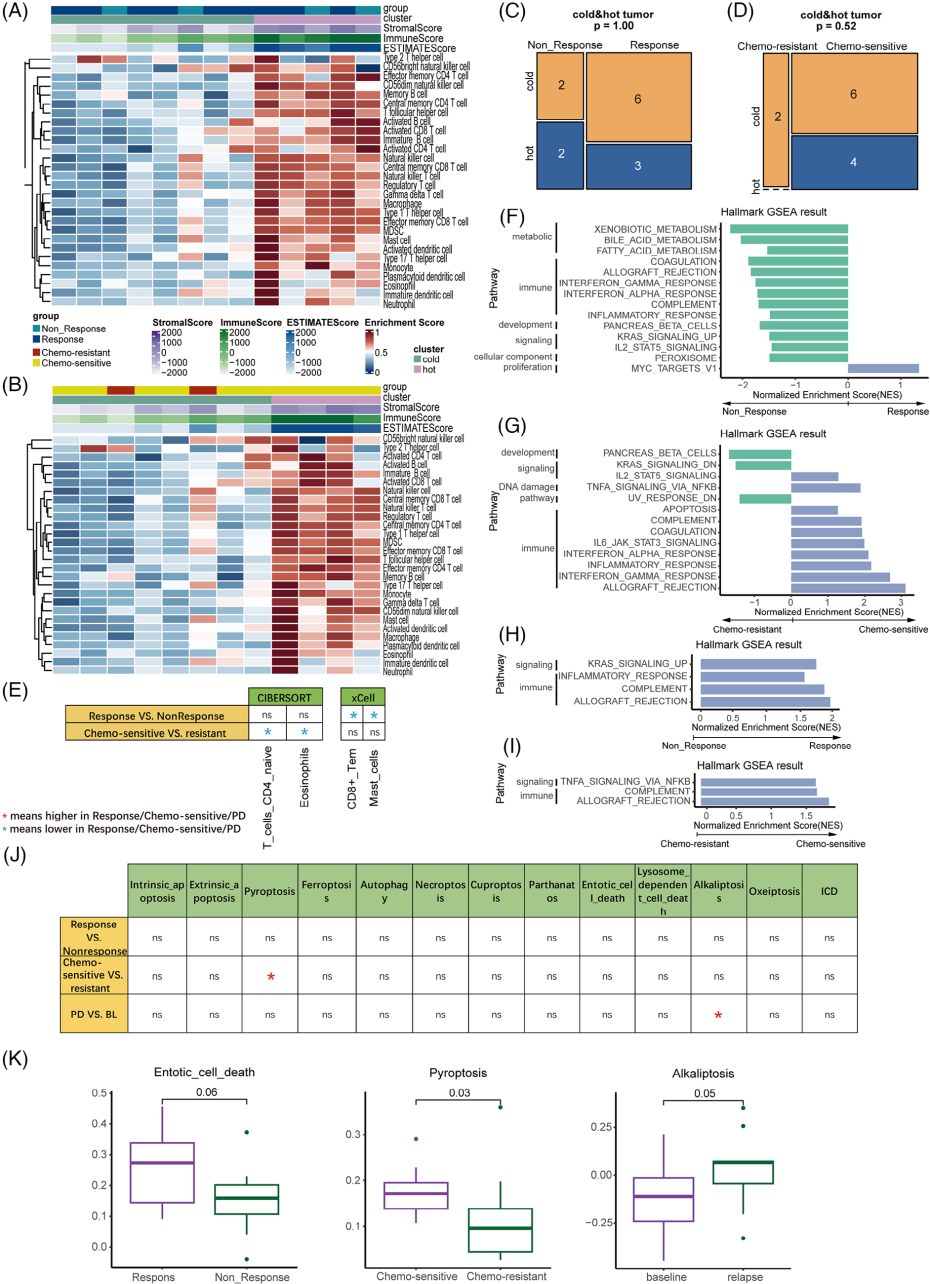
Association of immune cell infiltration, cell death and hallmark pathways with response and chemotherapy sensitivity in small cell lung cancer (SCLC) patients treated with chemotherapy. The heatmap shows 28 immune cells infiltration in response (A) and chemotherapy sensitivity (B). The bar plot shows immune hot and cold sample proportions in response (C) and chemotherapy sensitivity (D). (E) The table shows the distribution of immune cell infiltration between different responses and cohort patients, which shows the immune populations of CD8+ Tem and mast cells in non‐responders and CD4 naive T cells and eosinophils in chemo‐resistant were significantly higher. Hallmark pathways correlated the response (F and H) and chemotherapy sensitivity (G and I) in SCLC patients treated with chemotherapy. Cell death results for SCLC patients with significant differences in treatment response, chemotherapy sensitivity and/or treatment nodes are shown in (J). (K) Boxplots showing the enrichment scores of three cell death types (entotic cell death, pyroptosis and alkaliptosis) that showed significant differences in each of three groups.

After chemotherapy, the CTCs in chemo‐sensitive were significantly reduced at C3D1 while fewer changes were observed in chemo‐resistant (Figure S13). The changes in the CTC counts and molecular tumour burden index (mTBI)^5^ were nearly consistent during conventional follow‐up. The responders mostly tended to show a decreasing trend, while chemo‐resistant showed a more frequent increasing trend (Figure S14). Phylogenetic relation trees showed a sustained high cancer cell fraction of major clones in chemo‐resistant in both baseline and relapsed samples, but chemo‐sensitive was characterised by the weakening of major clones in baseline samples (Figure S15A,B). Although the average number of mutations of trunk private clones was significantly higher in non‐responders, the fraction of functional genes was lower (Figure S15C,D).

The genomic landscape of 21 paired baseline and relapsed ctDNA showed no significant differences in TMB and mTBI (Figure S16). The *KDR* gene was still one of the top 10 frequently mutated genes (Figure 3A). The platinum drug resistance pathway was significantly enriched in baseline subclonal mutations and relapsed clonal mutations (Figure 3B), which indicated that tumour with drug‐resistant mutations expanded from subclone to clone. Meanwhile, the tyrosine kinase inhibitor resistance and immune‐related pathways were enriched in relapsed clonal mutations and subclonal mutations, respectively. Finally, we summarised the correlation between mutation/pathway/immunity and pathological response or chemotherapy sensitivity (Figure 3C), which may provide a comprehensive concept of treatment response and resistance mechanisms in SCLC. Consistent global copy number variation (CNV) results from cell lines and patient 1022 were observed, and some significant CNV changes were found between patients with or without durable clinical benefit (Figure S17).

**FIGURE 3:**
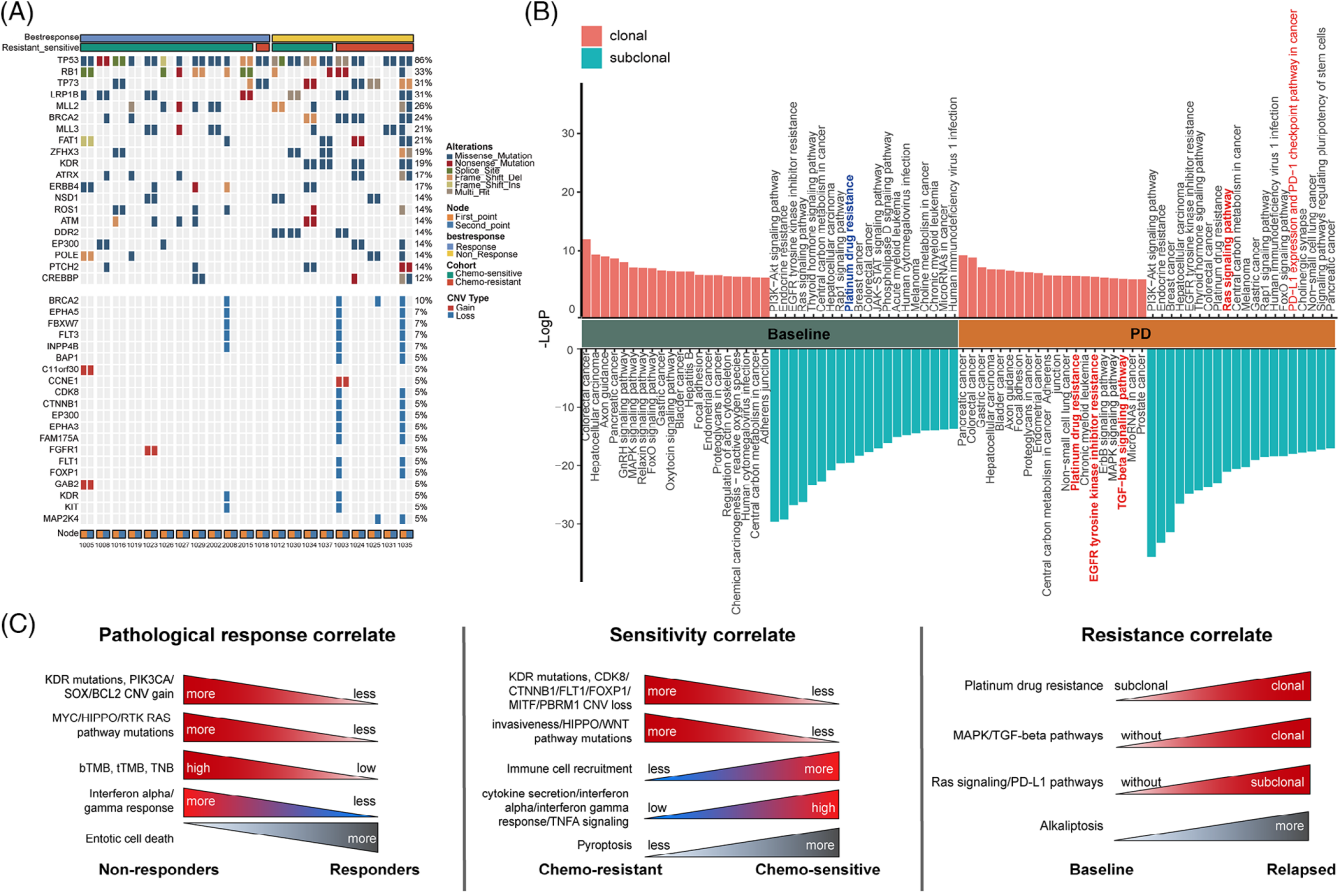
Genomic landscape and pathway analysis of paired baseline and relapsed circulating tumour DNA (ctDNA) samples in small cell lung cancer (SCLC). (A) Comparison of single nucleotide variations (SNVs) (upper) and copy number variations (CNVs) (lower) in paired baseline and relapsed ctDNA samples in SCLCs. The bar plot shows the proportion of response and chemotherapy sensitivity. (B) Clonal and subclonal gene enrichment pathways in baseline and PD samples. (C) The mechanism model diagram of mutational pathway in pathological response, chemotherapy sensitivity and resistance.

Patients with *KDR* mutation tended to have higher *KDR* expression levels and poor prognosis (Figure S18A,B). By using other datasets, we found that *KDR* was significantly highly expressed in SCLC‐I and patients with low expression of the *KDR* were enriched in SCLC‐A in the IMpower133 cohort^6^ (Figure S18C‒F). OS was significantly shorter in patients with high expression of *KDR* and VEGF pathways (Figure 4A,B). Moreover, the tumour with a high expression of *KDR* tended to be ‘hot’ (Figures 4C,D and S19). These findings were consistent with previous pathway enrichment results, suggesting that patients with *KDR* mutation and/or high expression of *KDR* may resist chemotherapy but benefit from immunotherapy, anti‐folates and AURK inhibitors.^7^ Several chemotherapy agents contained higher IC50 in the high *KDR* expression group (Figure 4E), which may reveal a resistant trend.

**FIGURE 4:**
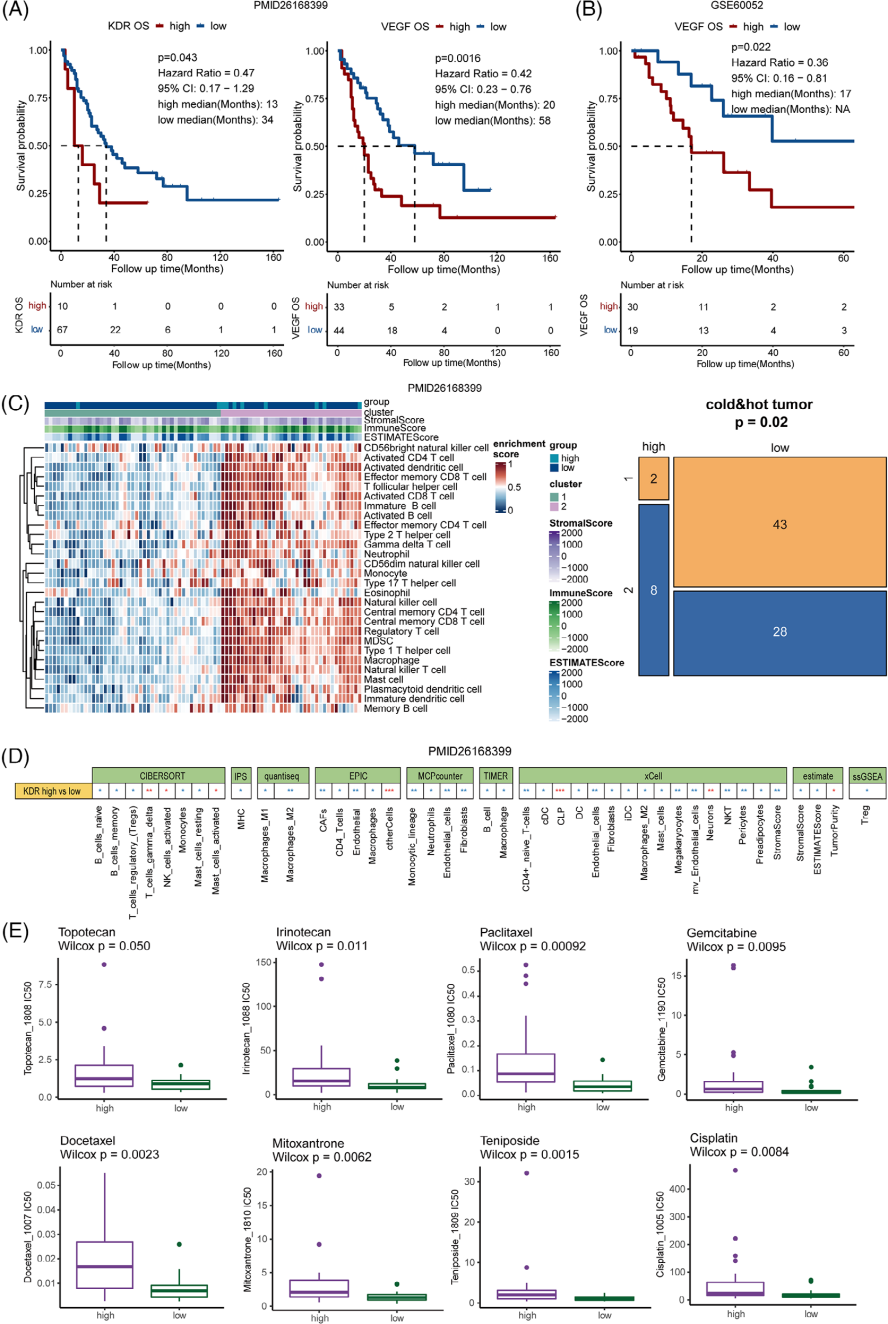
Survival and immune infiltration analysis of *KDR* and vascular endothelial growth factor (VEGF) expression in small cell lung cancer (SCLC). (A) Comparison of survival curves of low and high *KDR* and VEGF expression groups in the PMID: 26168399 dataset. (B) Comparison of survival curves of low and high VEGF expression groups in the GEO60052 dataset. (C) Tumour microenvironment (TME) analysis correlated low and high VEGF expression groups in the PMID: 26168399 dataset. (D) Heatmap showing the immune infiltration analysis results of CIBERSORT, IPS, Quantiseq, EPIC, MCPcounter, TIMER, xCell, Estimate and ssGSEA correlated with the low and high KDR expression groups in the GEO60052 dataset. (E) Boxplot of IC50 values for low and high KDR expression groups.

In conclusion, chemo‐sensitive/response patients showed beneficial survival, and we found the potential mechanism was that *KDR* mutation, *PI3K* amplification, VEGF and *KRAS* pathways activation contribute to the development of chemotherapy resistance. These results provide critical information for the clinical decision of VEGF signalling pathway inhibitors combined with chemotherapy and imply that targeted therapies may benefit some patients who are resistant to chemotherapy. Besides, the difference in tumour microenvironment and several immune pathways enrichment between chemo‐sensitive and chemo‐resistant was consistent with the concept that tumours can take control of environment to reset the body homeostasis.^8^ However, we still lack sufficient evidence to determine the most appropriate therapies for recurring patients. Future studies are warranted on larger cohorts of patients in a real‐world cohort to explore.

## AUTHOR CONTRIBUTIONS

*Conceptualisation, supervision, funding acquisition and writing—review and editing*: Ying Cheng. *Resources, data curation, software, formal analysis, methodology, writing—original draft and writing—review and editing*: Xuan Gao and Zelong Xu. *Formal analysis, methodology and writing—review and editing*: Bingfa Yan. *Conceptualisation, resources and writing—review and editing*: Jie Hu. *Resources, data curation and writing—review and editing*: Ying Liu, Jing Zhu, Ying Wang, Junfeng Wang, Changliang Yang, Hongxia Cui, Yanrong Wang, Guang Yang, Jie Hao, Peidong Li, Liang Zhang, Zili Li, Hongyu Wang, Yanli Sun, Shubo Zuo and Tianying Du. *Software, formal analysis and writing—review and editing*: Zhentian Liu, Xuefeng Xia and Xin Yi. *Resources and writing—review and editing*: Ying Xin, Ke Zheng, Yawen Yang and Kai Niu. *Formal analysis and writing—review and editing*: Jinhua Xu, Gan Zhang, Fei Chen and Ning Ding.

## CONFLICT OF INTEREST STATEMENT

Xuan Gao, Bingfa Yan, Zelong Xu, Zhentian Liu, Xuefeng Xia and Xin Yi are employees of Beijing GenePlus Technology Co., Ltd. The remaining authors declare that the research was conducted in the absence of any commercial or financial relationships that could be construed as potential conflicts of interest.

## ETHICS STATEMENT

All patients provided written informed consent to conduct research in this study, and ethical approvals were obtained from the two hospitals (NOPRODLUC0001). The study received approval to conduct genomic research from the China Human Genetic Resources Administration Office (HGRAO, 2016‐161).

## Supporting information

Supporting Information

Supporting Information

Supporting Information

Supporting Information

Supporting Information

Supporting Information

Supporting Information

Supporting Information

Supporting Information

Supporting Information

Supporting Information

Supporting Information

Supporting Information

Supporting Information

Supporting Information

Supporting Information

Supporting Information

Supporting Information

Supporting Information

Supporting Information

## Data Availability

All mutations reported in this study are provided in the Supporting Information. De‐identified patient clinical information is also provided in the Supporting information. Raw sequencing data have been deposited in the GSA‐Human database and accession number is HRA003770. All other relevant data could be obtained from the corresponding authors of this study.

## References

[1] Gazdar AF , Bunn PA , Minna JD . Small‐cell lung cancer: what we know, what we need to know and the path forward. Nat Rev Cancer. 2017;17:725‐737. doi:10.1038/nrc.2017.87 29077690

[2] Sabari JK , Lok BH , Laird JH , Poirier JT , Rudin CM . Unravelling the biology of SCLC: implications for therapy. Nat Rev Clin Oncol. 2017;14:549‐561. doi:10.1038/nrclinonc.2017.71 28534531 PMC5843484

[3] Bunn PA Jr , Minna JD , Augustyn A , et al. Small cell lung cancer: can recent advances in biology and molecular biology be translated into improved outcomes? J Thorac Oncol. 2016;11:453‐474. doi:10.1016/j.jtho.2016.01.012 26829312 PMC4836290

[4] Cullis J , Das S , Bar‐Sagi D . Kras and tumor immunity: friend or foe? Cold Spring Harb Perspect Med. 2018;8:a031849. doi:10.1101/cshperspect.a031849 29229670 PMC6120695

[5] Yi Z , Ma F , Rong G , et al. The molecular tumor burden index as a response evaluation criterion in breast cancer. Signal Transduct Target Ther. 2021;6:251. doi:10.1038/s41392-021-00662-9 34230452 PMC8260637

[6] Horn L , Mansfield AS , Szczesna A , et al. First‐line atezolizumab plus chemotherapy in extensive‐stage small‐cell lung cancer. N Engl J Med. 2018;379:2220‐2229. doi:10.1056/NEJMoa1809064 30280641

[7] Gay CM , Stewart CA , Park EM , et al. Patterns of transcription factor programs and immune pathway activation define four major subtypes of SCLC with distinct therapeutic vulnerabilities. Cancer Cell. 2021;39:346‐360. e347. doi:10.1016/j.ccell.2020.12.014 33482121 PMC8143037

[8] Slominski RM , Raman C , Chen JY , Slominski AT . How cancer hijacks the body's homeostasis through the neuroendocrine system. Trends Neurosci. 2023;46:263‐275. doi:10.1016/j.tins.2023.01.003 36803800 PMC10038913

