# Supplementary figures and images for: Multi‐omics molecular phenotyping reveals the potential mechanisms of chemotherapy response and resistance in small cell lung cancer
Source: Clin Transl Med. 2024 Jun 22;14(6):e1728. doi: 10.1002/ctm2.1728 (PMC11193134; doi:10.1002/ctm2.1728)

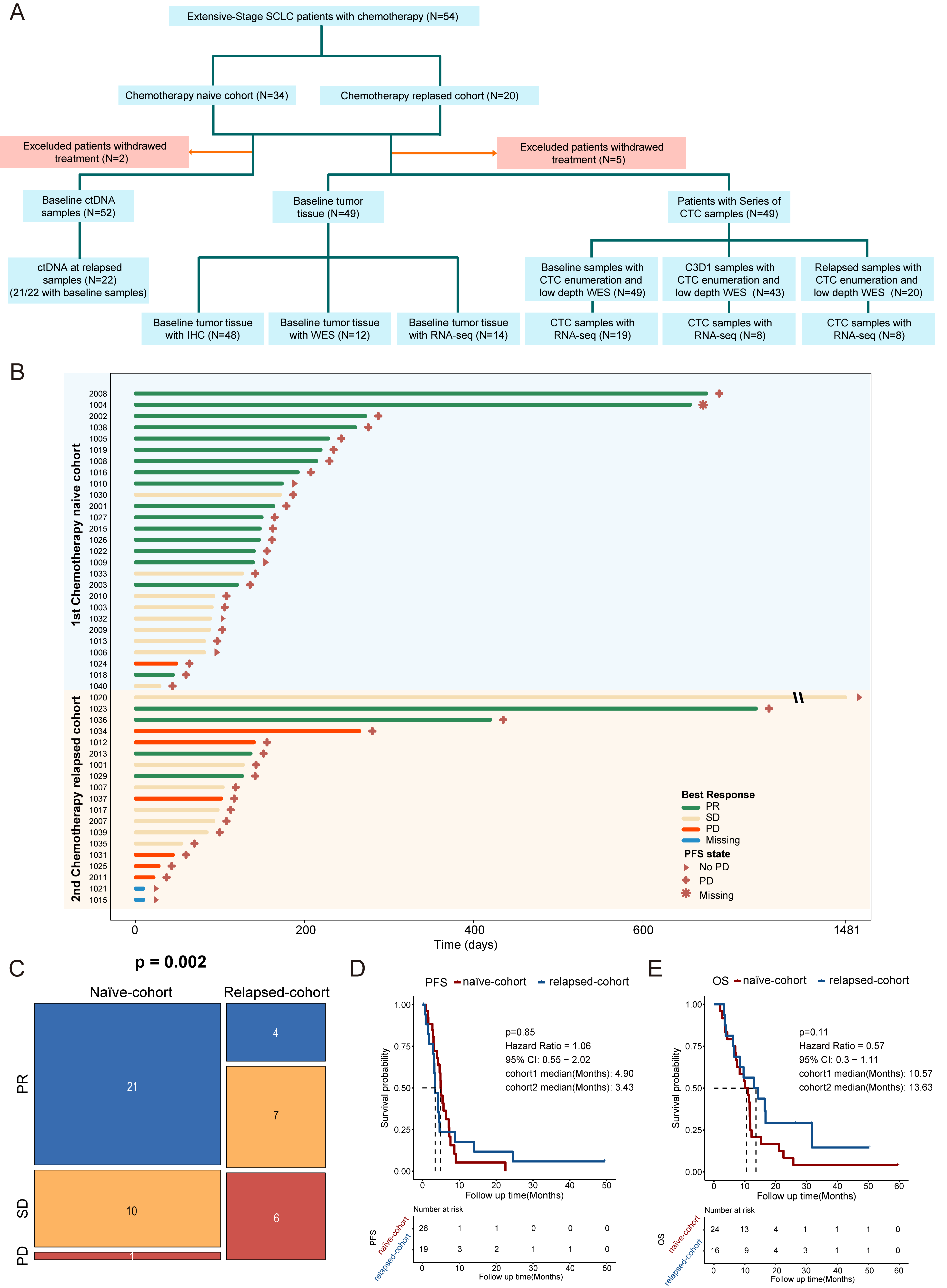

Supplement: Supplementary file 1 — Supporting Information [file CTM2-14-e1728-s006.tif]

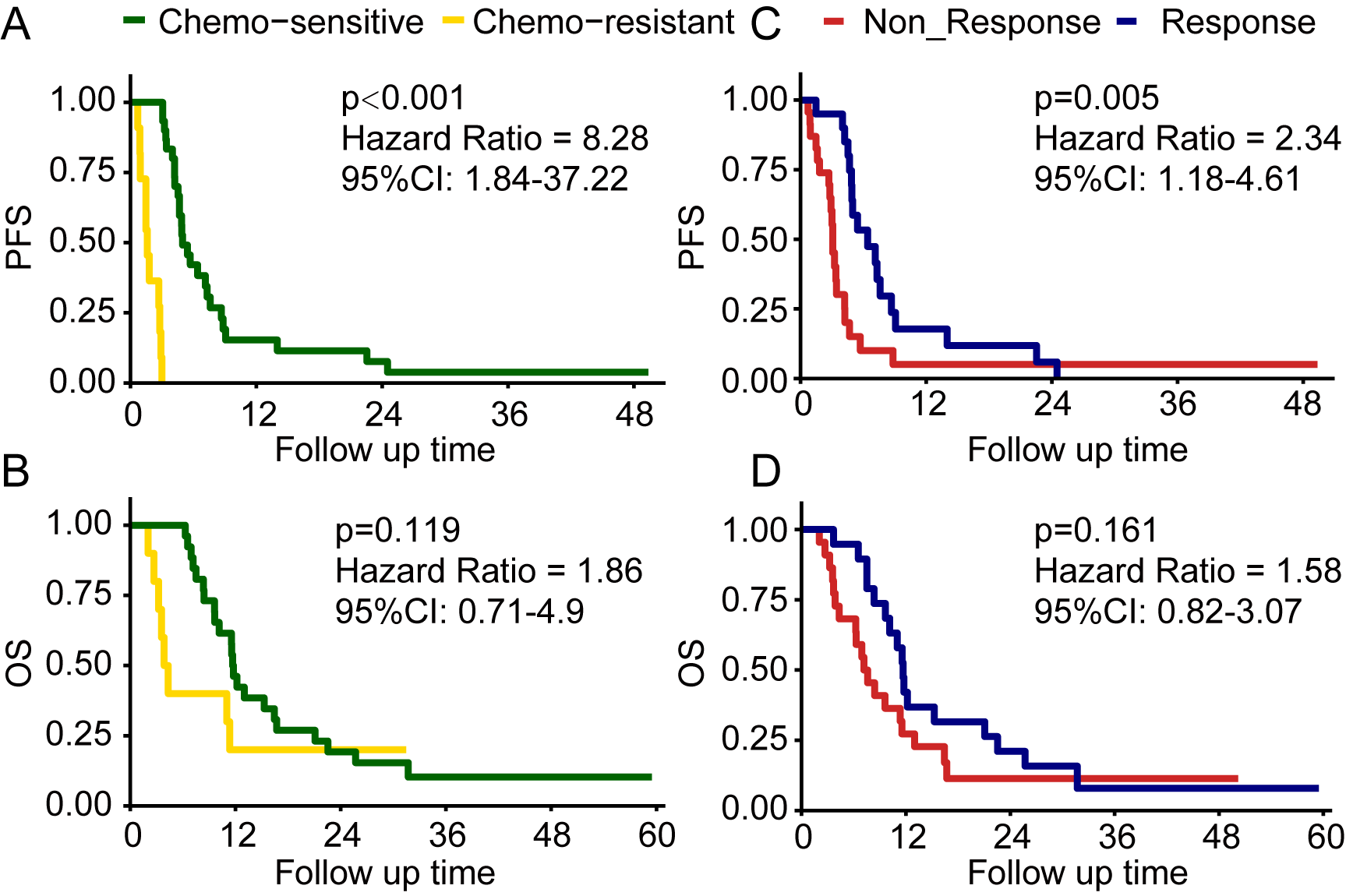

Supplement: Supplementary file 2 — Supporting Information [file CTM2-14-e1728-s003.tif]

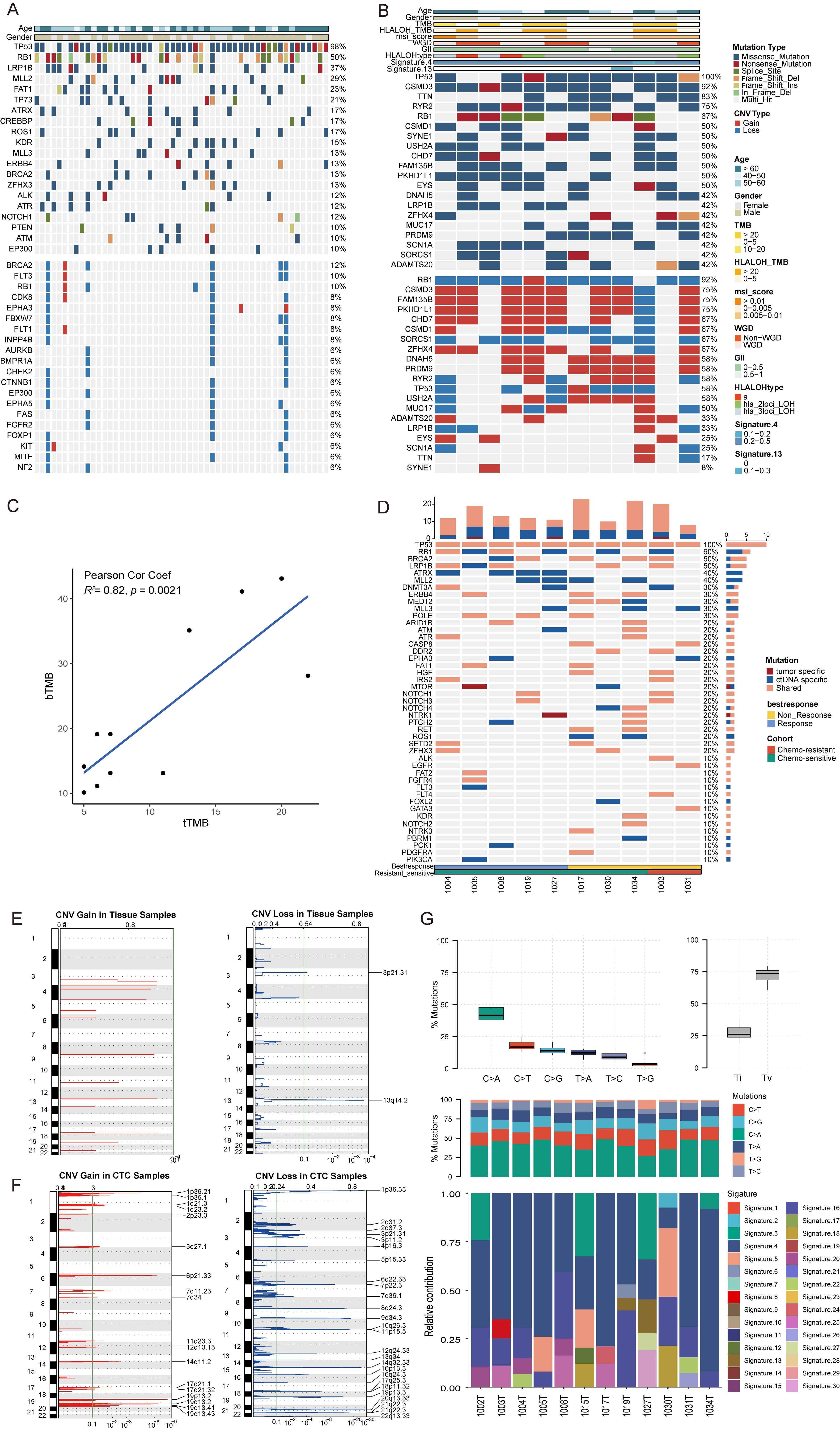

Supplement: Supplementary file 3 — Supporting Information [file CTM2-14-e1728-s020.tif]

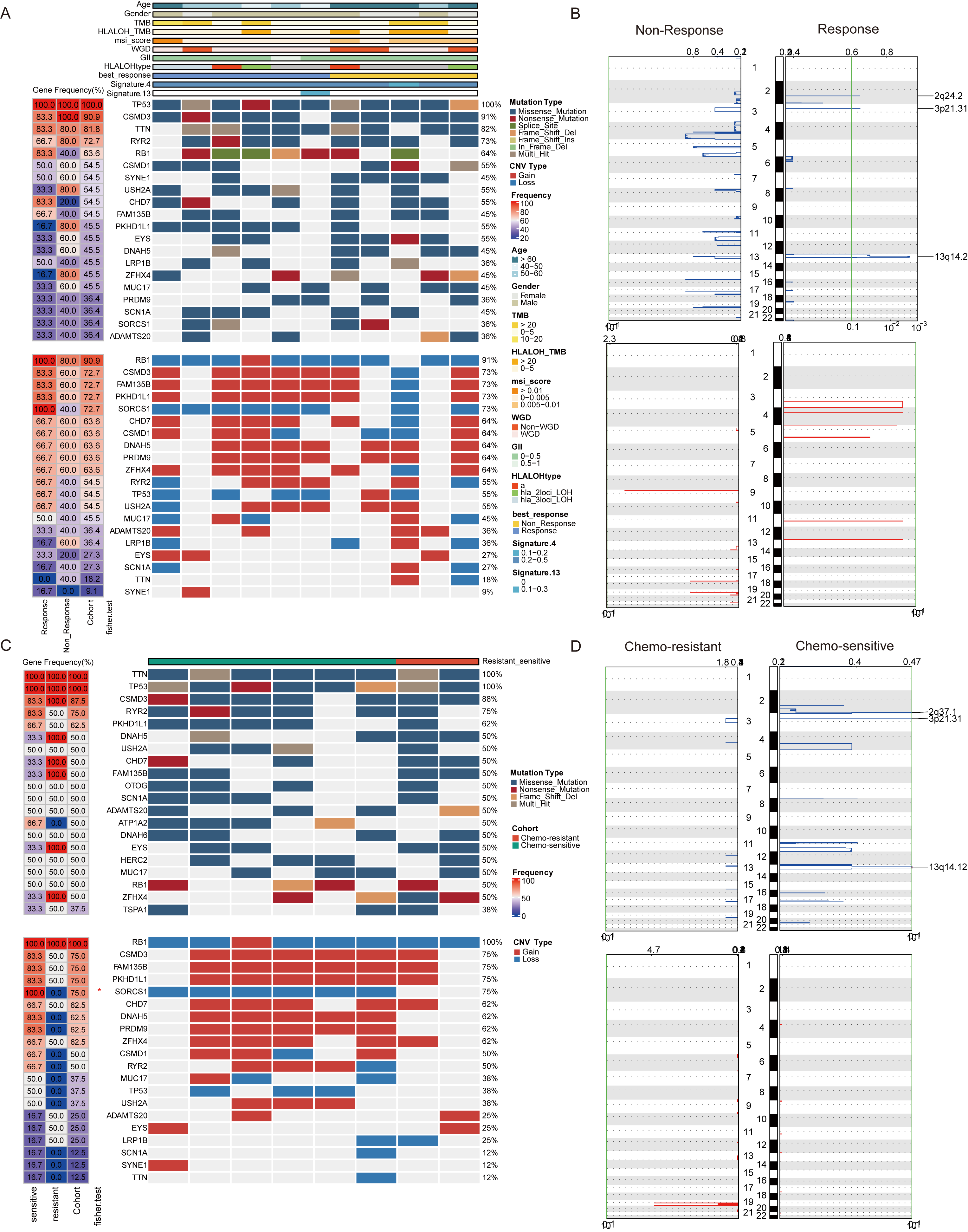

Supplement: Supplementary file 4 — Supporting Information [file CTM2-14-e1728-s019.tif]

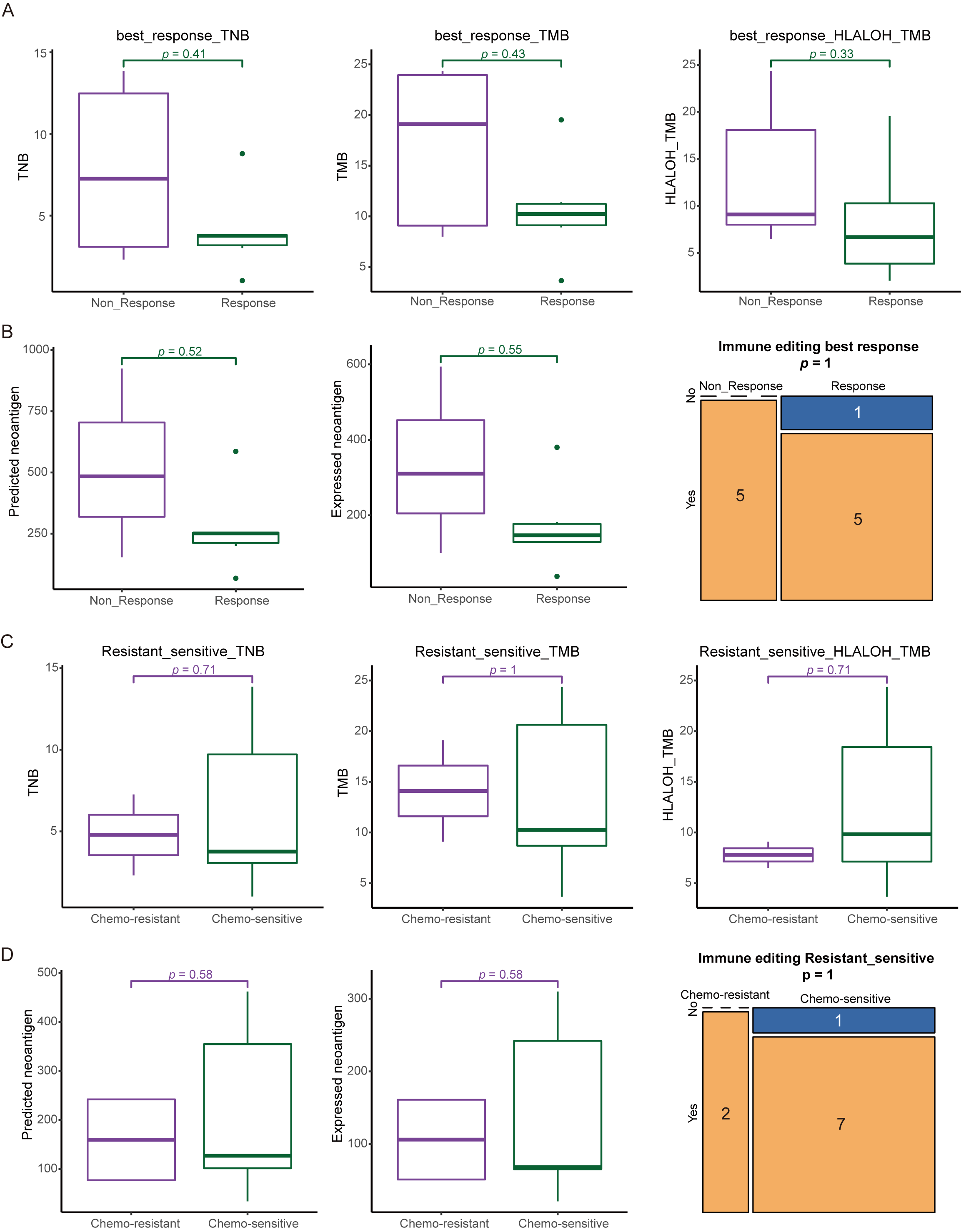

Supplement: Supplementary file 5 — Supporting Information [file CTM2-14-e1728-s008.tif]

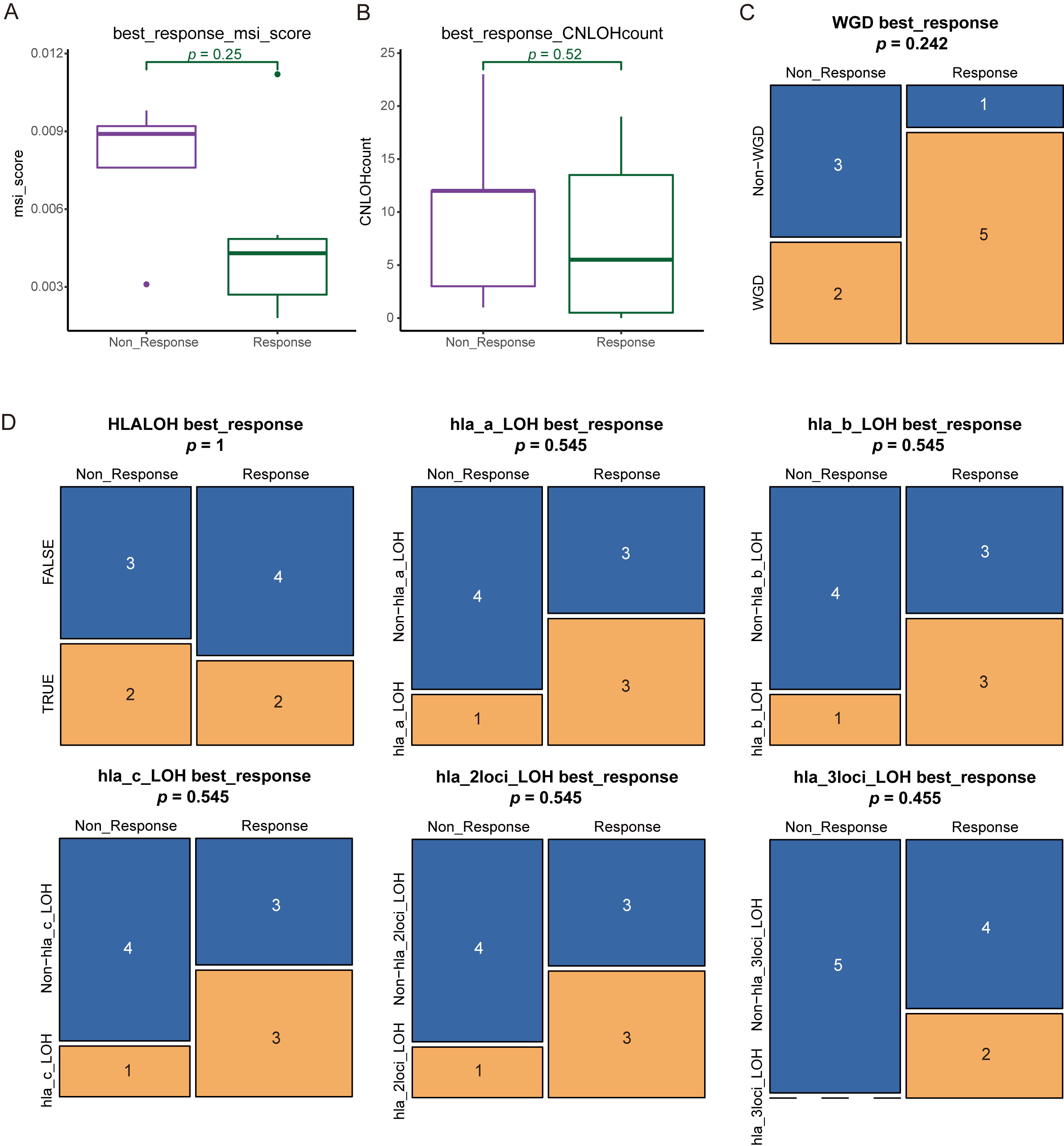

Supplement: Supplementary file 6 — Supporting Information [file CTM2-14-e1728-s011.tif]

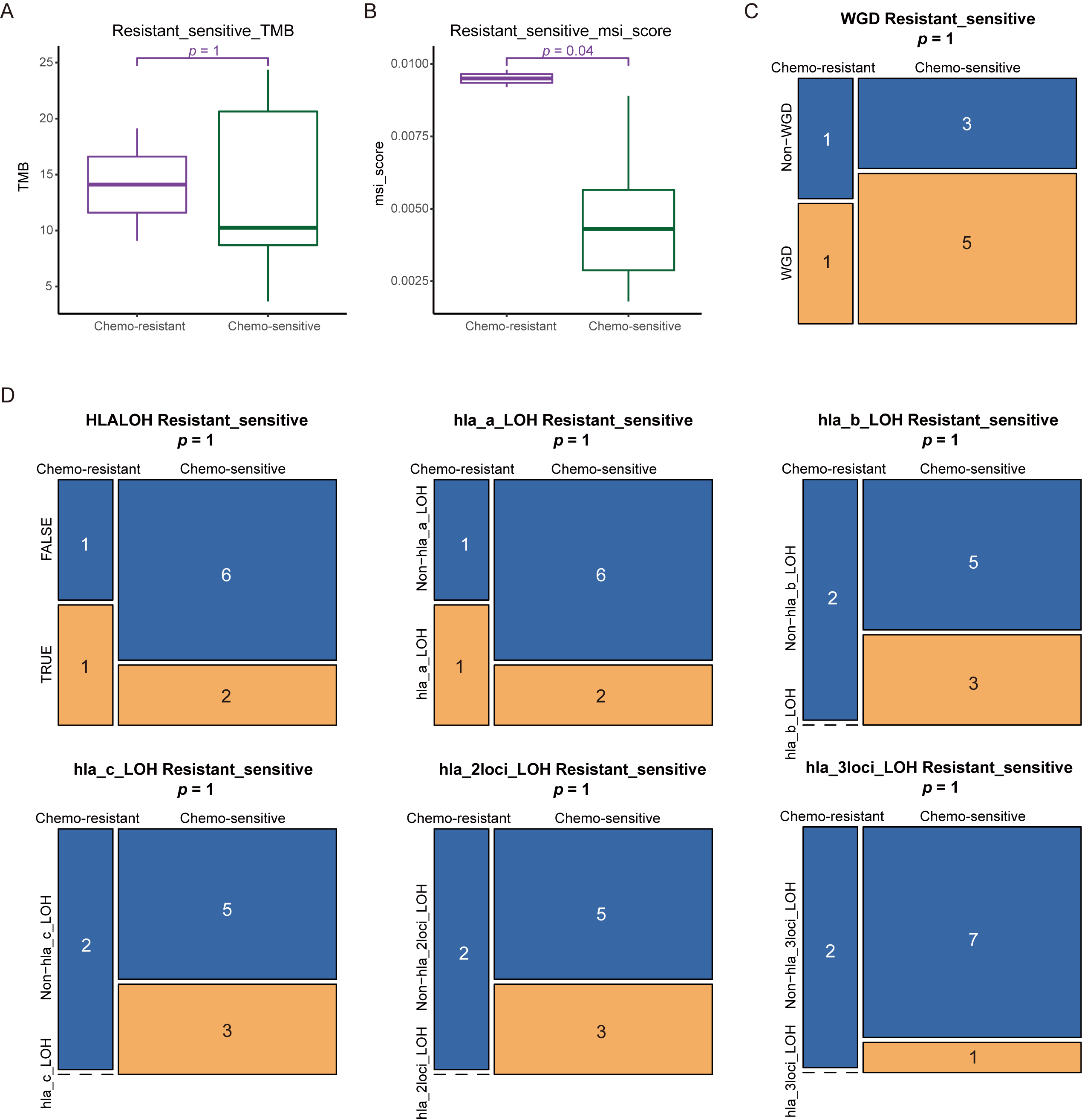

Supplement: Supplementary file 7 — Supporting Information [file CTM2-14-e1728-s015.tif]

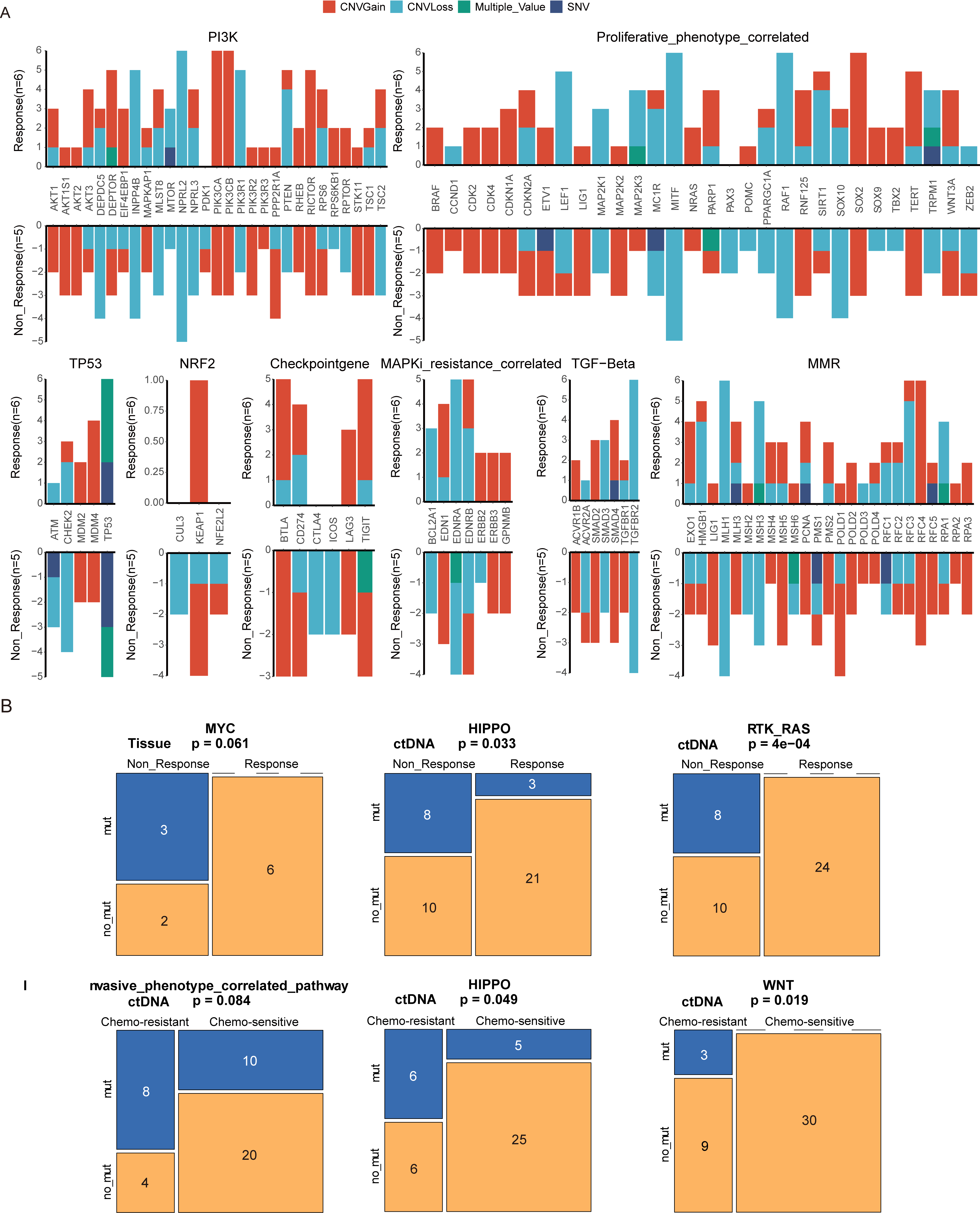

Supplement: Supplementary file 8 — Supporting Information [file CTM2-14-e1728-s014.tif]

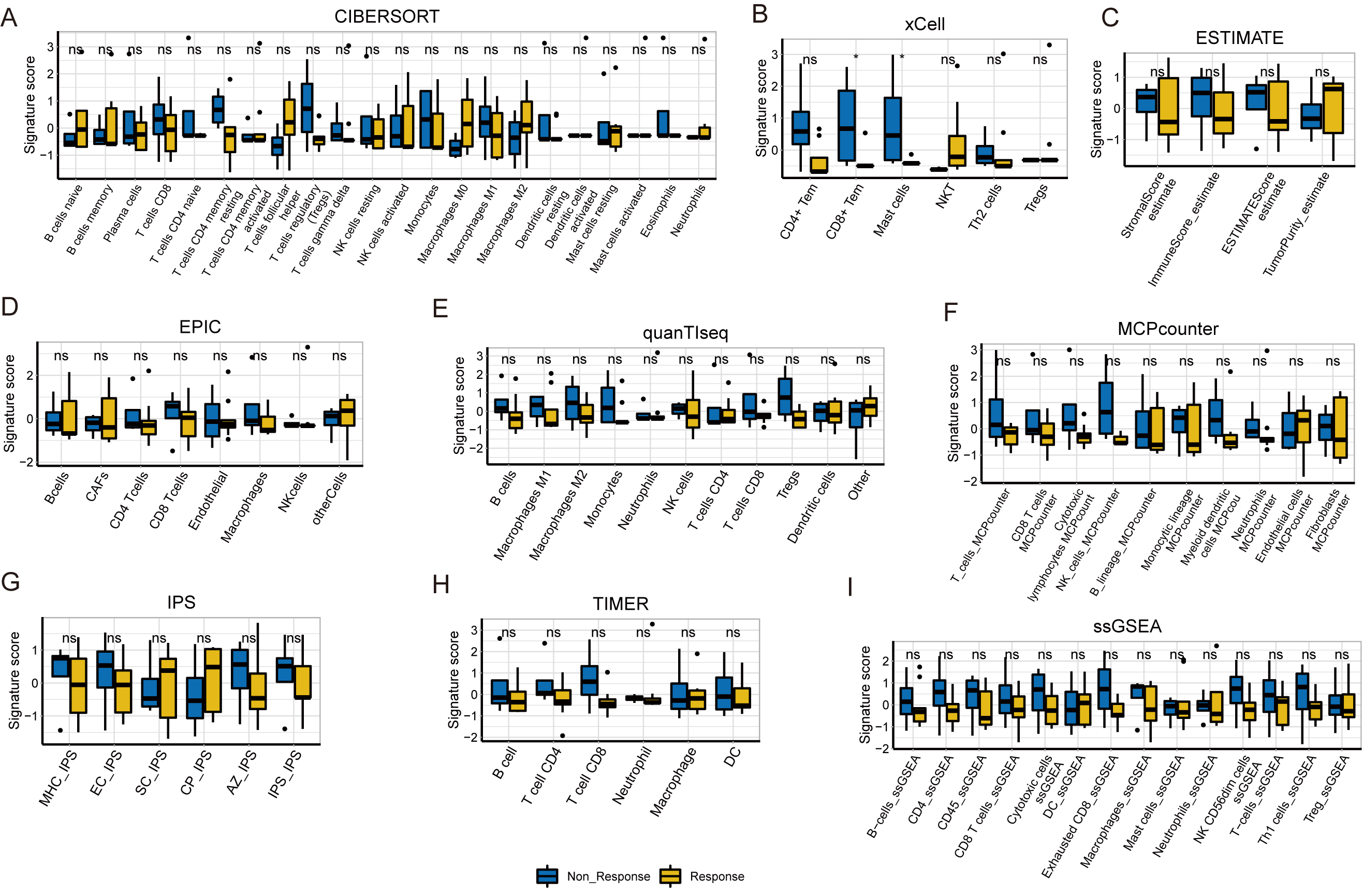

Supplement: Supplementary file 9 — Supporting Information [file CTM2-14-e1728-s007.tif]

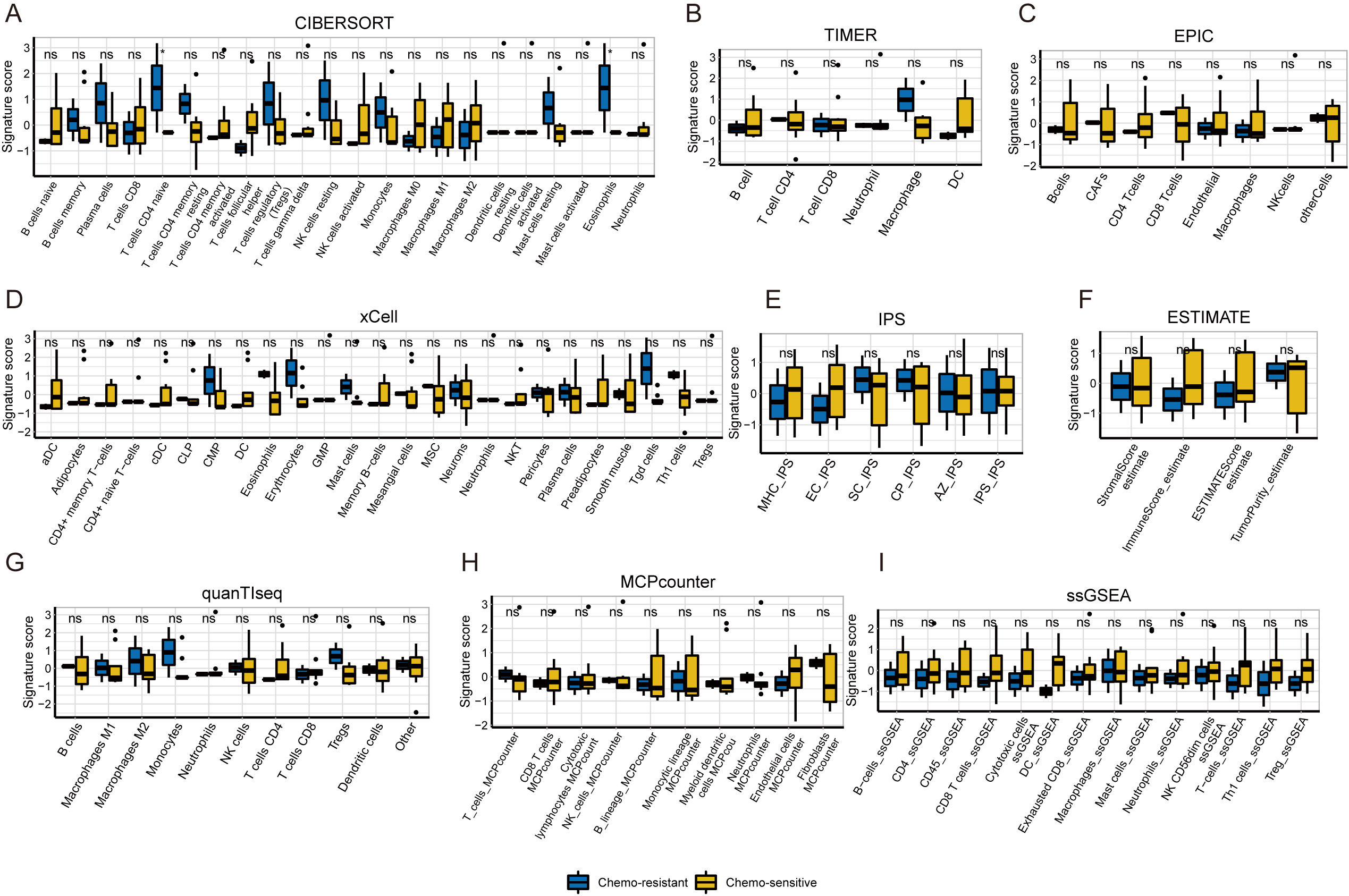

Supplement: Supplementary file 10 — Supporting Information [file CTM2-14-e1728-s005.tif]

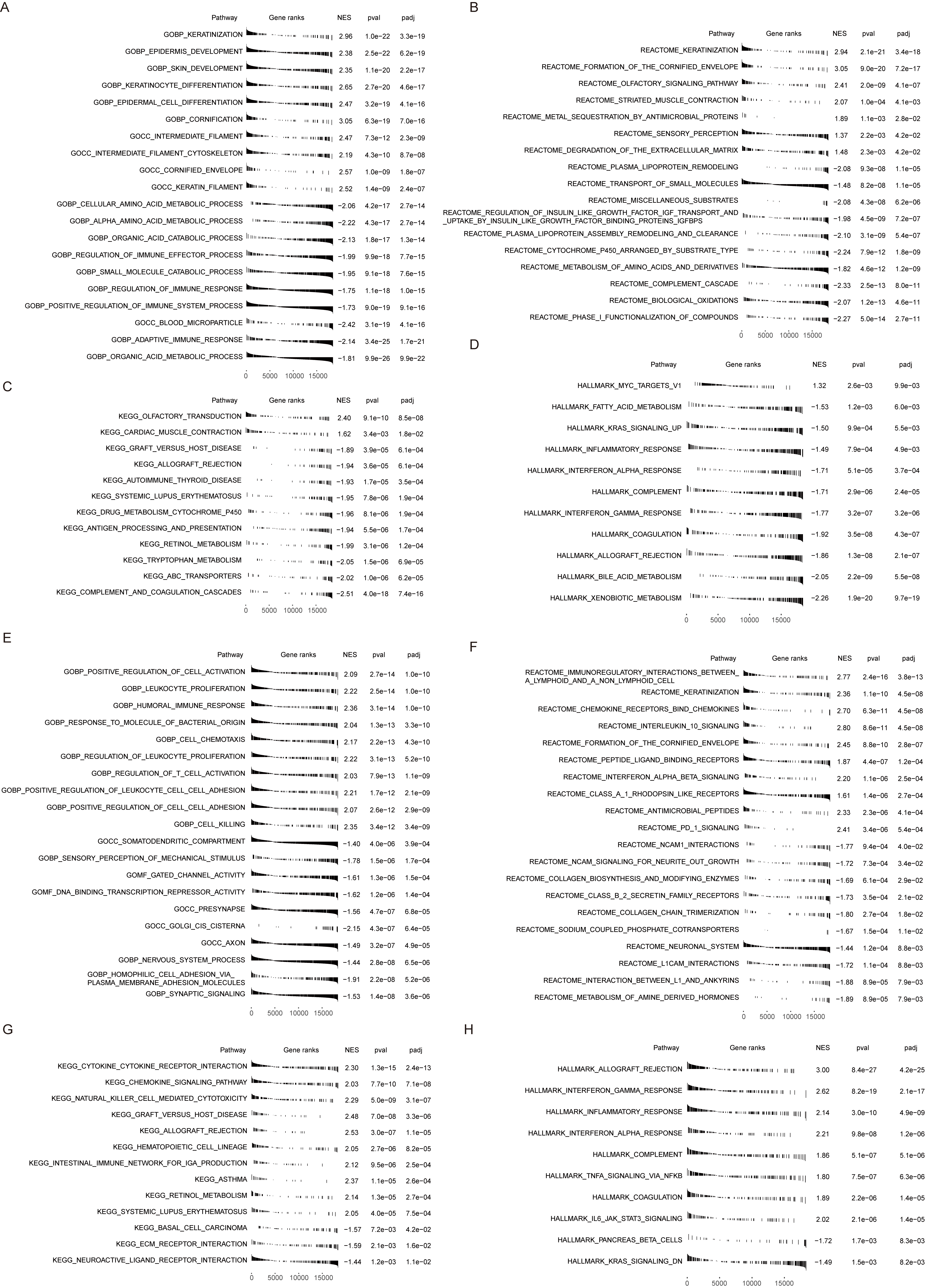

Supplement: Supplementary file 11 — Supporting Information [file CTM2-14-e1728-s001.tif]

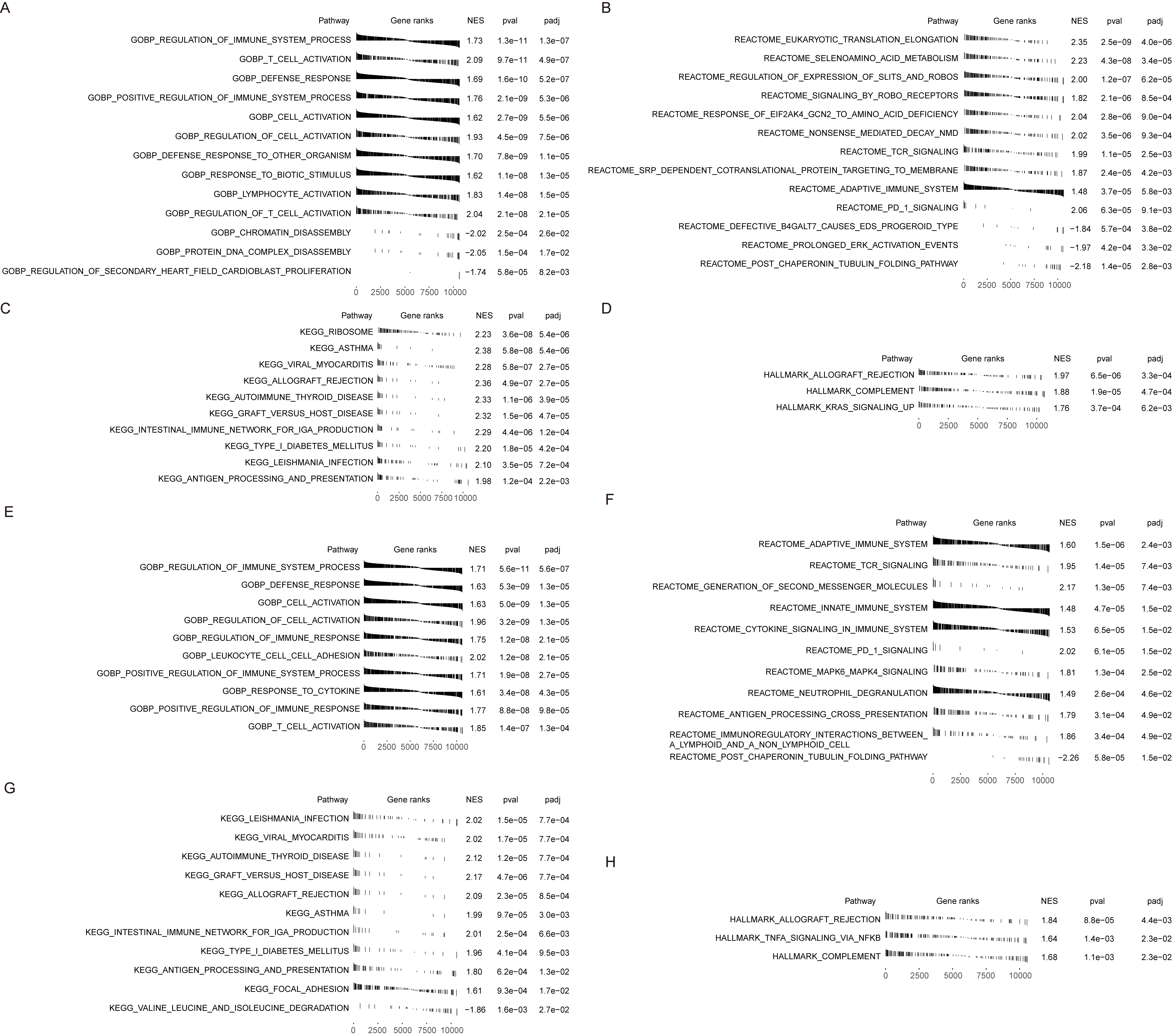

Supplement: Supplementary file 12 — Supporting Information [file CTM2-14-e1728-s012.tif]

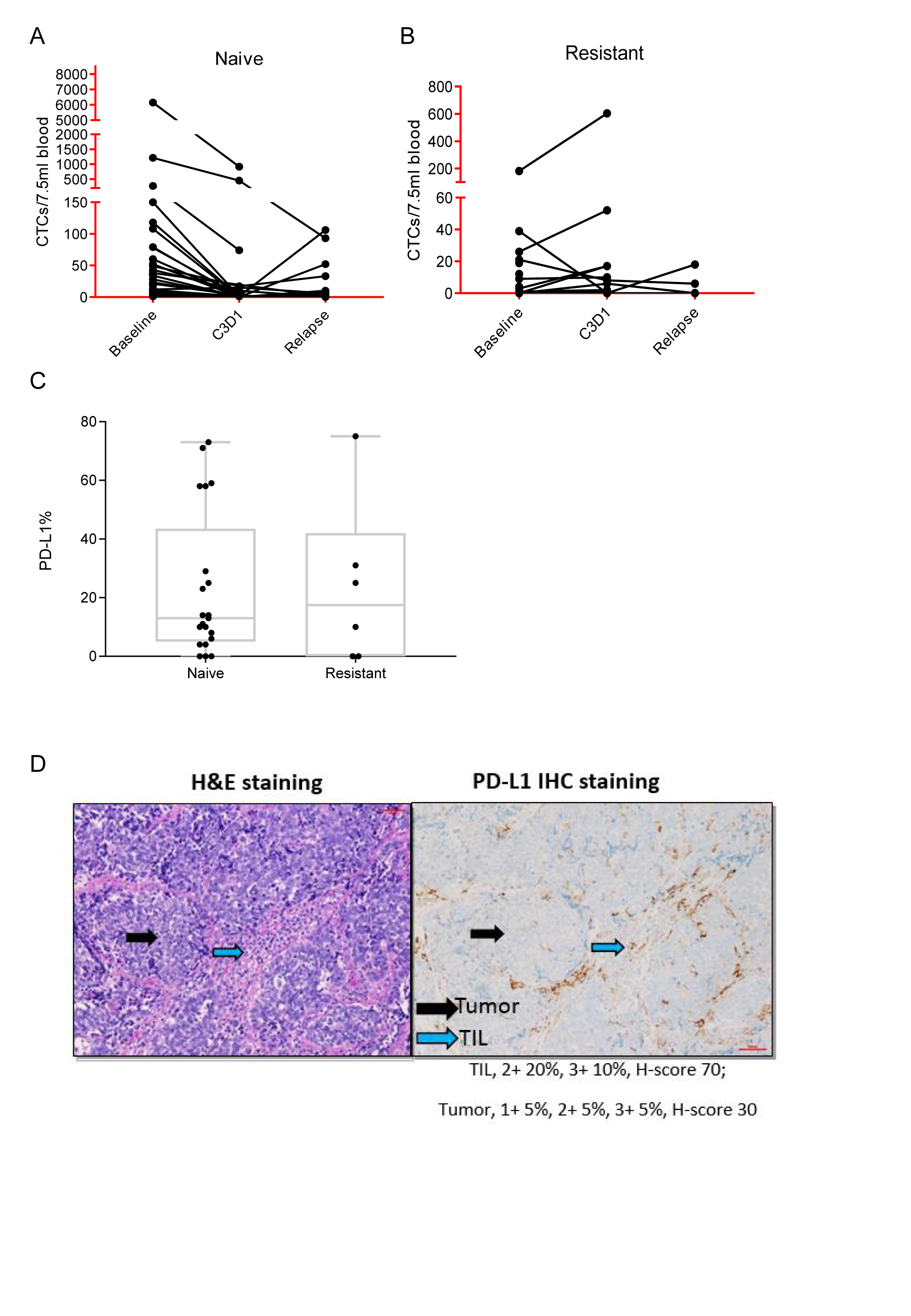

Supplement: Supplementary file 13 — Supporting Information [file CTM2-14-e1728-s002.tif]

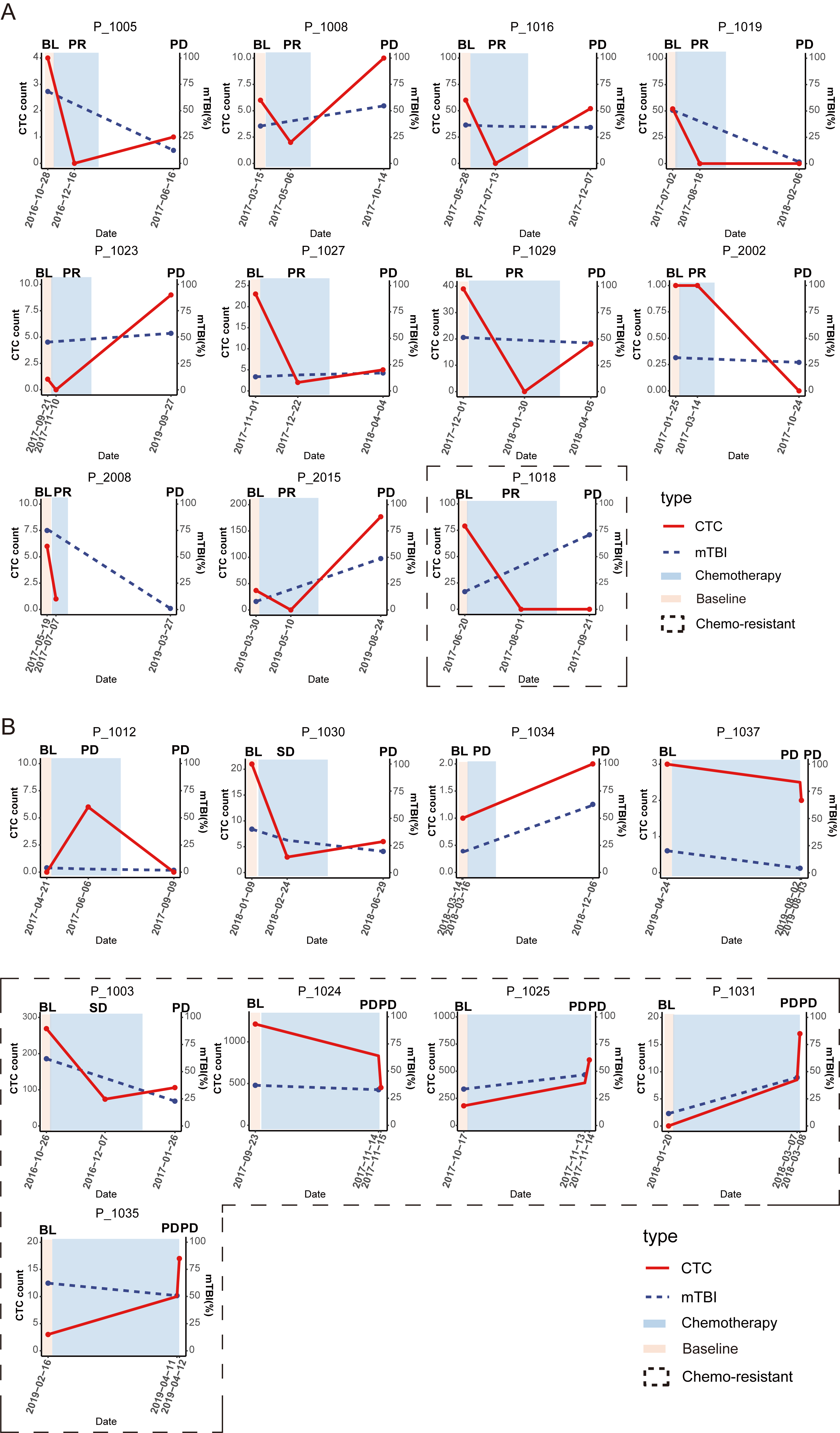

Supplement: Supplementary file 14 — Supporting Information [file CTM2-14-e1728-s018.tif]

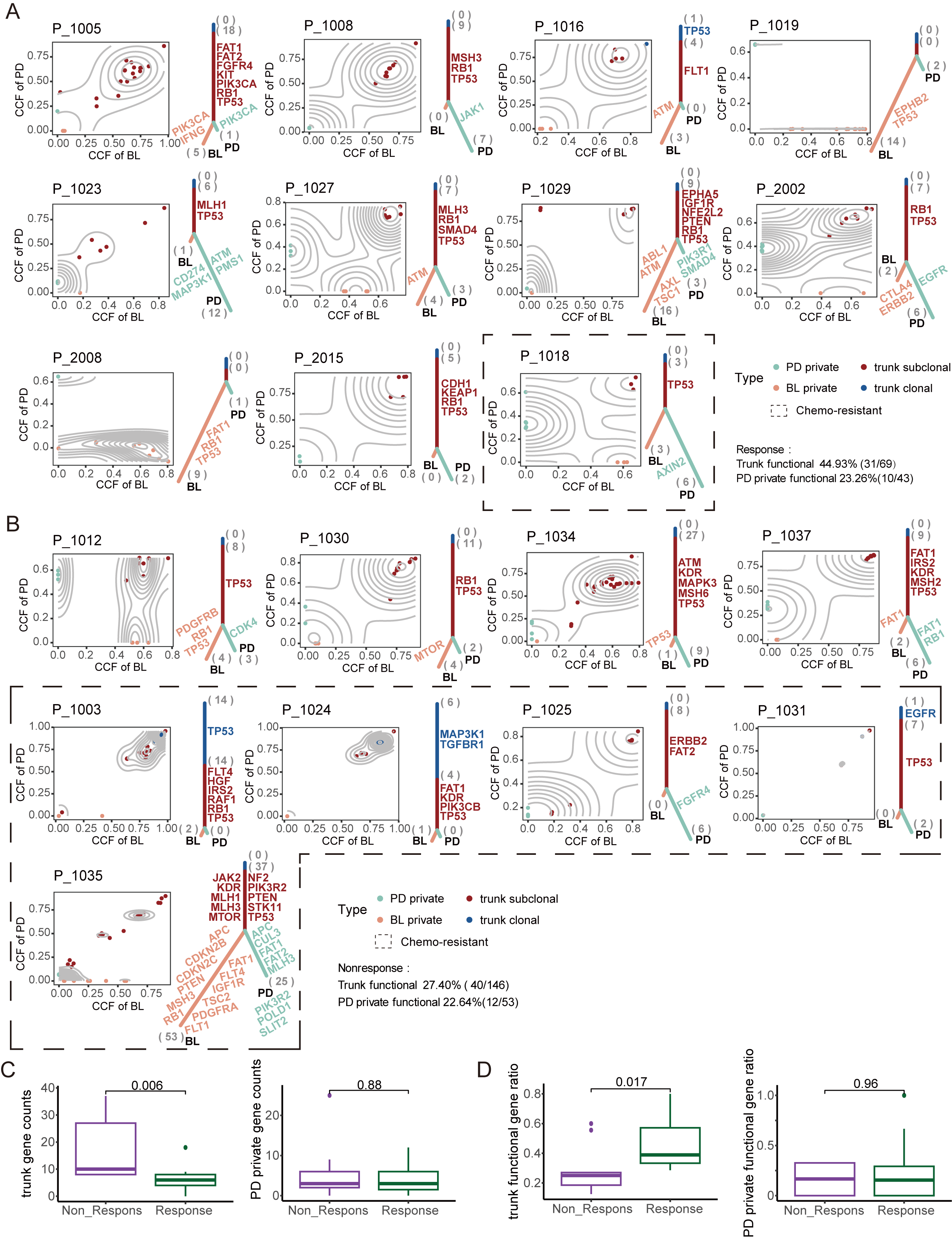

Supplement: Supplementary file 15 — Supporting Information [file CTM2-14-e1728-s009.tif]

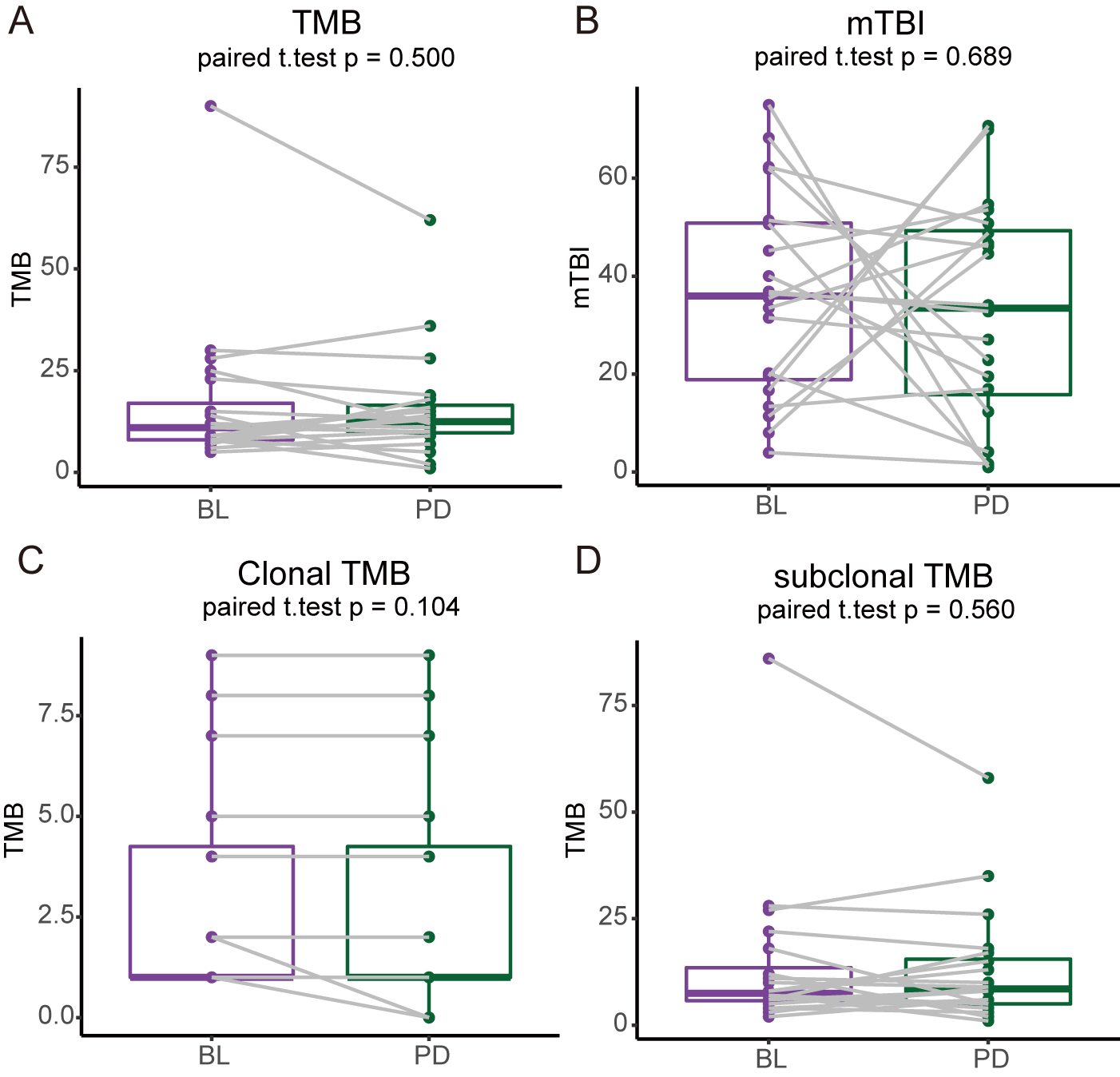

Supplement: Supplementary file 16 — Supporting Information [file CTM2-14-e1728-s013.tif]

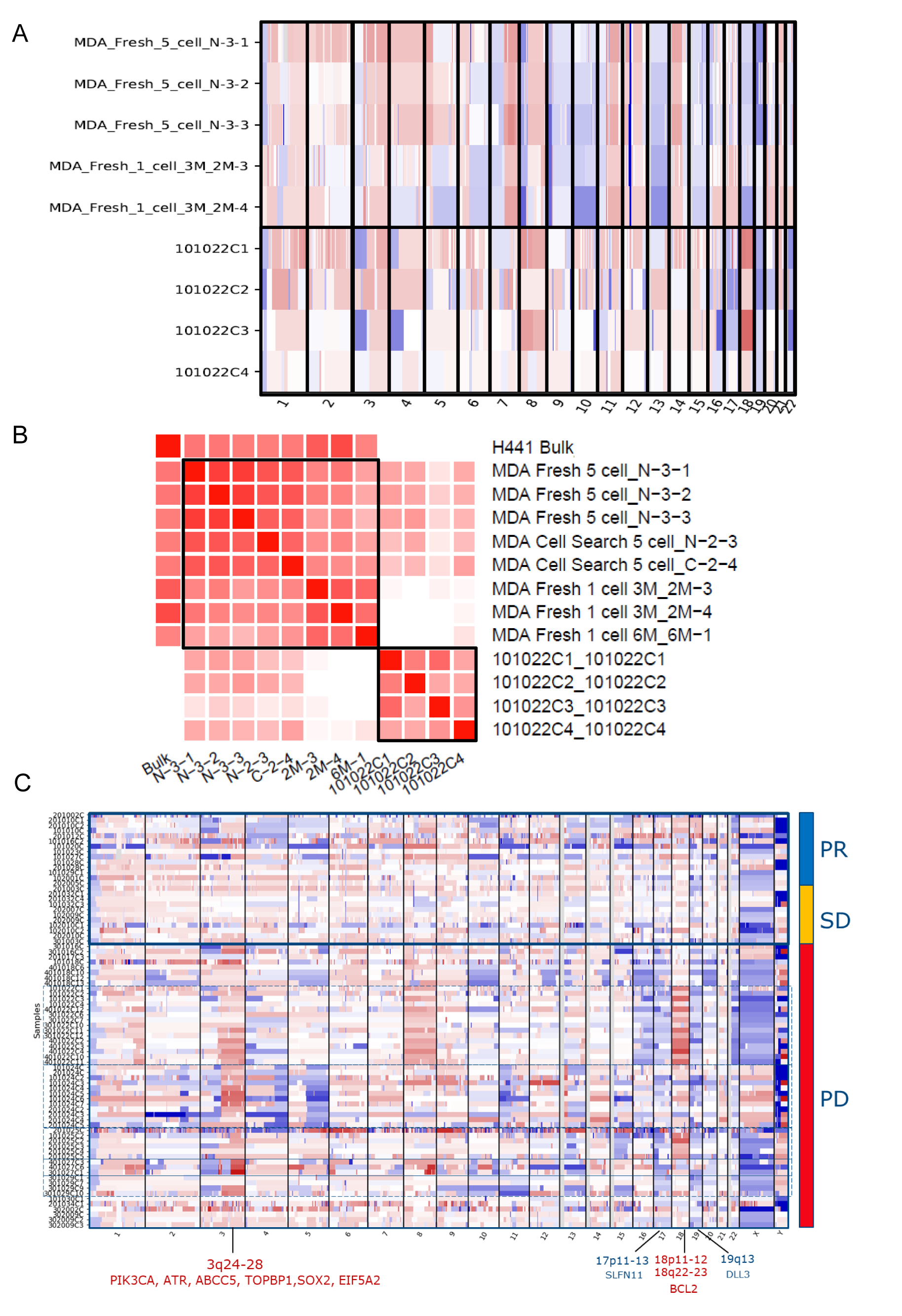

Supplement: Supplementary file 17 — Supporting Information [file CTM2-14-e1728-s004.tif]

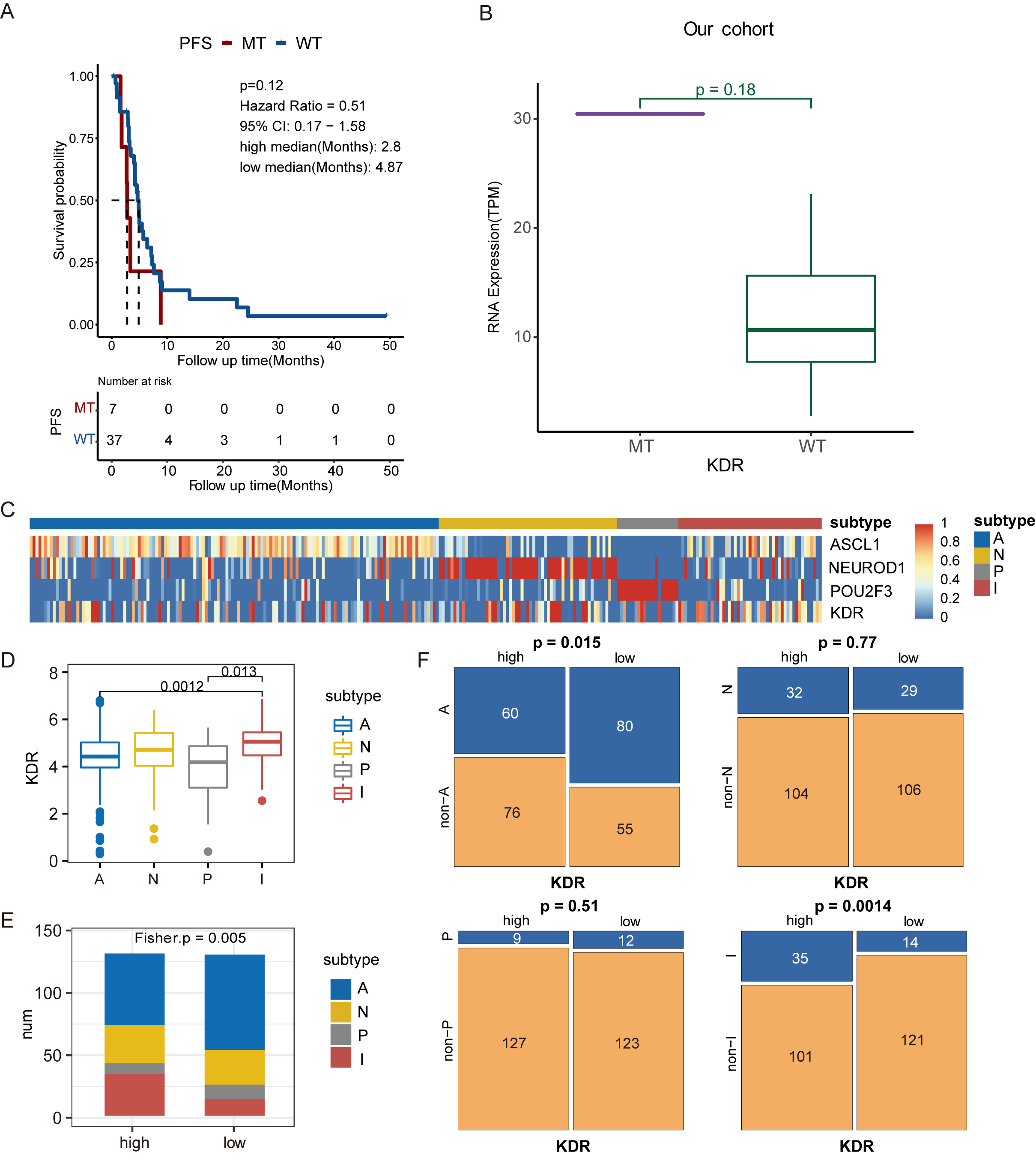

Supplement: Supplementary file 18 — Supporting Information [file CTM2-14-e1728-s010.tif]

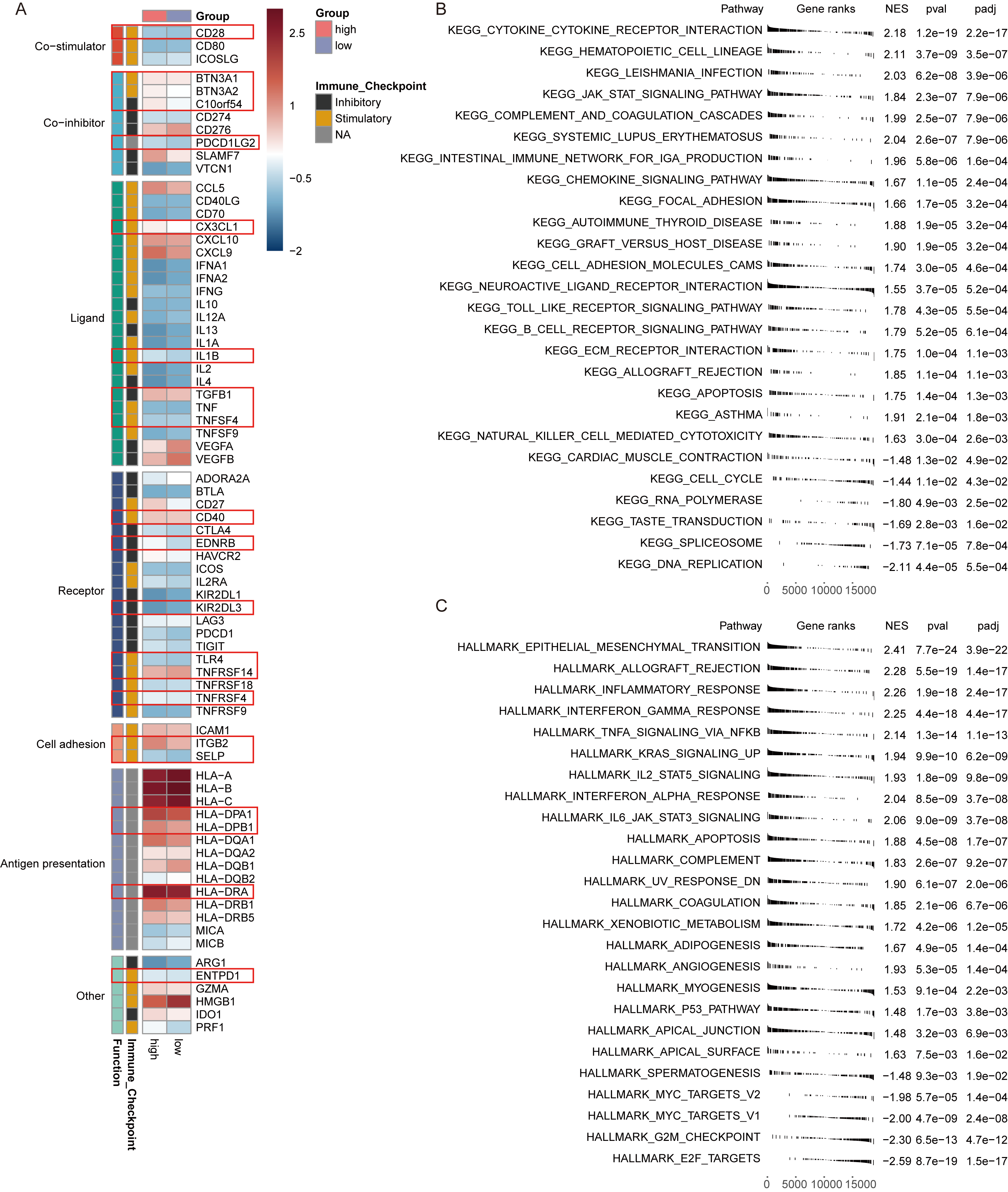

Supplement: Supplementary file 19 — Supporting Information [file CTM2-14-e1728-s017.tif]
