# Supplementary material for: Multi‐omics molecular phenotyping reveals the potential mechanisms of chemotherapy response and resistance in small cell lung cancer
Source: Clin Transl Med. 2024 Jun 22;14(6):e1728. doi: 10.1002/ctm2.1728 (PMC11193134; doi:10.1002/ctm2.1728)
Supplement: Supplementary file 20 — Supporting Information [file CTM2-14-e1728-s016.docx]

Table S1. Baseline characteristics of enrolled SCLC patients and comparison of baseline characteristics between two groups.

| Characteristics | Chemotherapy naive subjects N=34 | 2nd line of chemotherapy resistant or refractory subjects N=20 | P Value | Total (%) |
| --- | --- | --- | --- | --- |
| Sex |  |  | 0.771 |  |
| Male | 22 | 14 |  | 36 (66.67%) |
| Female | 12 | 6 |  | 18 (33.33%) |
| Age |  |  | 0.161 |  |
| <60 | 13 | 12 |  | 25 (46.30%) |
| >=60 | 21 | 8 |  | 29 (53.70%) |
| AJCC TNM Stage at Initial Diagnosis |  |  | 0.011 |  |
| I | 0 | 0 |  | 0 |
| II | 0 | 1 |  | 1 (1.85%) |
| IIIa | 1 | 4 |  | 5 (9.26%) |
| IIIb | 1 | 3 |  | 4 (7.41%) |
| IV | 31 | 12 |  | 43 (79.63%) |
| Unknown | 1 | 0 |  | 1 (1.85%) |
| Prior Radiotherapy |  |  | < 0.0001 |  |
| Yes | 1 | 10 |  | 11 (20.37%) |
| No | 33 | 10 |  | 43 (79.63%) |
| First-line treatment information |  |  | 0.725 |  |
| EC | 9 | 7 |  | 16 (29.63%) |
| EC+Lobaplatin | 0 | 1 |  | 1 (1.85%) |
| EP | 20 | 10 |  | 30 (55.56%) |
| EP+Temozolomide | 1 | 0 |  | 1 (1.85%) |
| Etoposide | 1 | 0 |  | 1 (1.85%) |
| Etoposide+DDP+Temozolomide | 1 | 0 |  | 1 (1.85%) |
| IP | 1 | 1 |  | 2 (3.71%) |
| Temozolomide | 0 | 1 |  | 1 (1.85%) |
| Temozolomide+Atezolizumab | 1 | 0 |  | 1 (1.85%) |

Abbreviation: EC, Etoposide+Cisplatin; EP, Etoposide+Carboplatin; DDP, Cisplatin; IP, Etoposide+Carboplatin.

Table S2. Univariate cox regression for overall survival/progression-free survival based on different clinical parameters.

|  | **Parameter** | **P-value** | **Hazard Ratio** | **95% Confidence Interval** |
| --- | --- | --- | --- | --- |
| **OS** | Sex (Male vs. Female) | 0.838 | 1.07 | 0.56-2.06 |
|  | Age | 0.475 | 1.02 | 0.97-1.06 |
|  | Stage (IV vs. II-III) | 0.389 | 1.47 | 0.61-3.53 |
| **PFS** | Sex (Male vs. Female) | 0.891 | 0.95 | 0.47-1.91 |
|  | Age | 0.557 | 0.99 | 0.95-1.03 |
|  | Stage (IV vs. II-III) | 0.0984 | 1.99 | 0.88-4.49 |

Table S3. ctDNA specific mutations detected from ctDNA analysis which were compared with WES data from 12 matched baseline tumor samples.

| ID | Gene | type | ID | Gene | type |
| --- | --- | --- | --- | --- | --- |
| 1002 | TP53 | ctDNA specific | 1003 | TP53 | ctDNA specific |
| 1002 | ATR | ctDNA specific | 1005 | ERBB4 | ctDNA specific |
| 1002 | APC | ctDNA specific | 1005 | POLE | ctDNA specific |
| 1002 | CDK12 | ctDNA specific | 1005 | RB1 | ctDNA specific |
| 1002 | ATRX | ctDNA specific | 1005 | SLX4 | ctDNA specific |
| 1002 | RARA | ctDNA specific | 1005 | TP53 | ctDNA specific |
| 1002 | FAT2 | ctDNA specific | 1005 | TOP2A | ctDNA specific |
| 1002 | RARA | ctDNA specific | 1005 | BCOR | ctDNA specific |
| 1002 | PDGFRB | ctDNA specific | 1005 | AR | ctDNA specific |
| 1002 | DNMT3A | ctDNA specific | 1005 | FANCM | ctDNA specific |
| 1002 | ROS1 | ctDNA specific | 1005 | FAT2 | ctDNA specific |
| 1003 | HDAC4 | ctDNA specific | 1005 | FGFR4 | ctDNA specific |
| 1003 | BRCA2 | ctDNA specific | 1005 | ESR1 | ctDNA specific |
| 1003 | RARA | ctDNA specific | 1005 | KIT | ctDNA specific |
| 1003 | TP53 | ctDNA specific | 1005 | FAT1 | ctDNA specific |
| 1003 | RAF1 | ctDNA specific | 1005 | PIK3CA | ctDNA specific |
| 1003 | TP53 | ctDNA specific | 1005 | PIK3CA | ctDNA specific |
| 1003 | DDR2 | ctDNA specific | 1019 | EPHB2 | ctDNA specific |
| 1003 | ABL2 | ctDNA specific | 1019 | MLL2 | ctDNA specific |
| 1003 | HGF | ctDNA specific | 1019 | CDC73 | ctDNA specific |
| 1003 | RB1 | ctDNA specific | 1019 | NOTCH1 | ctDNA specific |
| 1003 | NOTCH1 | ctDNA specific | 1019 | MLL2 | ctDNA specific |
| 1003 | LRP1B | ctDNA specific | 1019 | SMARCB1 | ctDNA specific |
| 1003 | LRP1B | ctDNA specific | 1019 | ATRX | ctDNA specific |
| 1003 | NOTCH3 | ctDNA specific | 1019 | BRCA2 | ctDNA specific |
| 1003 | ALK | ctDNA specific | 1019 | POLE | ctDNA specific |
| 1003 | ALK | ctDNA specific | 1019 | NOTCH3 | ctDNA specific |
| 1003 | FLT4 | ctDNA specific | 1019 | TP53 | ctDNA specific |
| 1003 | GRIN2A | ctDNA specific | 1027 | RB1 | ctDNA specific |
| 1003 | IRS2 | ctDNA specific | 1027 | TP53 | ctDNA specific |
| 1003 | DNMT3A | ctDNA specific | 1027 | ATRX | ctDNA specific |
| 1003 | CTCF | ctDNA specific | 1027 | ATM | ctDNA specific |
| 1003 | ELAC2 | ctDNA specific | 1027 | MLL3 | ctDNA specific |
| 1004 | SETD2 | ctDNA specific | 1027 | MLH3 | ctDNA specific |
| 1004 | DNMT3A | ctDNA specific | 1027 | SMAD4 | ctDNA specific |
| 1004 | LRP1B | ctDNA specific | 1027 | MLL2 | ctDNA specific |
| 1004 | TP53 | ctDNA specific | 1027 | SMO | ctDNA specific |
| 1004 | TP53 | ctDNA specific | 1027 | DNMT3A | ctDNA specific |
| 1004 | ATRX | ctDNA specific | 1008 | LRP1B | ctDNA specific |
| 1004 | DAXX | ctDNA specific | 1008 | EPHA3 | ctDNA specific |
| 1004 | MLL | ctDNA specific | 1008 | MSH3 | ctDNA specific |
| 1004 | RB1 | ctDNA specific | 1008 | ARID1B | ctDNA specific |
| 1004 | ATR | ctDNA specific | 1008 | PTCH2 | ctDNA specific |
| 1004 | HSP90AA1 | ctDNA specific | 1008 | JAK1 | ctDNA specific |
| 1004 | ZFHX3 | ctDNA specific | 1008 | TP53 | ctDNA specific |
| 1004 | IRS2 | ctDNA specific | 1008 | GNA11 | ctDNA specific |
| 1005 | ERBB4 | ctDNA specific | 1008 | BRCA2 | ctDNA specific |
| 1005 | LRP1B | ctDNA specific | 1008 | RB1 | ctDNA specific |
| 1005 | IFNG | ctDNA specific | 1008 | ATRX | ctDNA specific |
| 1005 | POLE | ctDNA specific | 1008 | PCK1 | ctDNA specific |
| 1005 | FLT3 | ctDNA specific | 1008 | EP300 | ctDNA specific |
| 1005 | PIK3CA | ctDNA specific | 1030 | LRP1B | ctDNA specific |
| 1005 | PIK3CA | ctDNA specific | 1030 | LRP1B | ctDNA specific |
| 1005 | KIT | ctDNA specific | 1030 | ARID1A | ctDNA specific |
| 1005 | FAT1 | ctDNA specific | 1030 | DDR2 | ctDNA specific |
| 1005 | FAT2 | ctDNA specific | 1030 | MED12 | ctDNA specific |
| 1005 | FGFR4 | ctDNA specific | 1030 | TP53 | ctDNA specific |
| 1005 | ESR1 | ctDNA specific | 1030 | ZFHX3 | ctDNA specific |
| 1005 | AR | ctDNA specific | 1030 | RB1 | ctDNA specific |
| 1005 | SLX4 | ctDNA specific | 1030 | FOXL2 | ctDNA specific |
| 1005 | TP53 | ctDNA specific | 1030 | MTOR | ctDNA specific |
| 1005 | RB1 | ctDNA specific | 1030 | MED12 | ctDNA specific |
| 1005 | FANCM | ctDNA specific | 1031 | GATA3 | ctDNA specific |
| 1005 | TOP2A | ctDNA specific | 1031 | MLL3 | ctDNA specific |
| 1005 | BCOR | ctDNA specific | 1031 | CYLD | ctDNA specific |
| 1008 | RB1 | ctDNA specific | 1031 | EPHA3 | ctDNA specific |
| 1008 | TP53 | ctDNA specific | 1031 | EGFR | ctDNA specific |
| 1008 | LRP1B | ctDNA specific | 1031 | TP53 | ctDNA specific |
| 1008 | EP300 | ctDNA specific | 1031 | CASP8 | ctDNA specific |
| 1008 | ARID1B | ctDNA specific | 1031 | SMARCA4 | ctDNA specific |
| 1008 | MSH3 | ctDNA specific | 1019 | PPM1D | ctDNA specific |
| 1015 | PHF6 | ctDNA specific | 1019 | FAM123B | ctDNA specific |
| 1015 | FLCN | ctDNA specific | 1034 | NOTCH4 | ctDNA specific |
| 1015 | FLT1 | ctDNA specific | 1034 | C11orf30 | ctDNA specific |
| 1015 | RB1 | ctDNA specific | 1034 | NTRK1 | ctDNA specific |
| 1015 | FAT1 | ctDNA specific | 1034 | BRCA2 | ctDNA specific |
| 1015 | TP53 | ctDNA specific | 1034 | KDR | ctDNA specific |
| 1015 | ALK | ctDNA specific | 1034 | TP53 | ctDNA specific |
| 1015 | EPHA5 | ctDNA specific | 1034 | CIC | ctDNA specific |
| 1015 | LRP1B | ctDNA specific | 1034 | RET | ctDNA specific |
| 1017 | TP53 | ctDNA specific | 1034 | SERPINB4 | ctDNA specific |
| 1017 | MLL2 | ctDNA specific | 1034 | MSH6 | ctDNA specific |
| 1017 | AXIN2 | ctDNA specific | 1034 | ATR | ctDNA specific |
| 1017 | CTNNA1 | ctDNA specific | 1034 | NOTCH2 | ctDNA specific |
| 1017 | PMS1 | ctDNA specific | 1034 | TP73 | ctDNA specific |
| 1017 | POLE | ctDNA specific | 1034 | PBRM1 | ctDNA specific |
| 1017 | KDM6A | ctDNA specific | 1034 | ERBB4 | ctDNA specific |
| 1017 | RAD51D | ctDNA specific | 1034 | TP53 | ctDNA specific |
| 1017 | MED12 | ctDNA specific | 1034 | PTCH2 | ctDNA specific |
| 1017 | CASP8 | ctDNA specific | 1034 | ATM | ctDNA specific |
| 1017 | MED12 | ctDNA specific | 1034 | ARID1B | ctDNA specific |
| 1017 | NTRK3 | ctDNA specific | 1031 | EGFR | ctDNA specific |
| 1017 | PMS1 | ctDNA specific | 1031 | CASP8 | ctDNA specific |
| 1017 | BRCA2 | ctDNA specific | 1031 | TP53 | ctDNA specific |
| 1017 | PDGFRA | ctDNA specific | 1031 | CYLD | ctDNA specific |
| 1017 | ROS1 | ctDNA specific | 1031 | MLL3 | ctDNA specific |
| 1017 | MED12 | ctDNA specific | 1031 | GATA3 | ctDNA specific |
| 1017 | BARD1 | ctDNA specific | 1030 | DDR2 | ctDNA specific |
| 1017 | ERBB4 | ctDNA specific | 1030 | LRP1B | ctDNA specific |
| 1017 | AXIN1 | ctDNA specific | 1030 | LRP1B | ctDNA specific |
| 1017 | CTNNA1 | ctDNA specific | 1030 | NOTCH4 | ctDNA specific |
| 1017 | HRAS | ctDNA specific | 1030 | RB1 | ctDNA specific |
| 1017 | ERBB4 | ctDNA specific | 1030 | ZFHX3 | ctDNA specific |
| 1017 | RET | ctDNA specific | 1030 | TP53 | ctDNA specific |
| 1017 | HGF | ctDNA specific | 1030 | MED12 | ctDNA specific |
| 1017 | HNF1A | ctDNA specific | 1034 | NTRK1 | ctDNA specific |
| 1017 | FAT1 | ctDNA specific | 1034 | C11orf30 | ctDNA specific |
| 1017 | SETD2 | ctDNA specific | 1034 | KDR | ctDNA specific |
| 1003 | ABL2 | ctDNA specific | 1034 | PTCH2 | ctDNA specific |
| 1003 | DDR2 | ctDNA specific | 1034 | ARID1B | ctDNA specific |
| 1003 | RAF1 | ctDNA specific | 1034 | PBRM1 | ctDNA specific |
| 1003 | FLT4 | ctDNA specific | 1034 | ATM | ctDNA specific |
| 1003 | HGF | ctDNA specific | 1034 | ATR | ctDNA specific |
| 1003 | NOTCH1 | ctDNA specific | 1034 | BRCA2 | ctDNA specific |
| 1003 | ALK | ctDNA specific | 1034 | ERBB4 | ctDNA specific |
| 1003 | ALK | ctDNA specific | 1034 | TP73 | ctDNA specific |
| 1003 | LRP1B | ctDNA specific | 1034 | NOTCH2 | ctDNA specific |
| 1003 | LRP1B | ctDNA specific | 1034 | MLL3 | ctDNA specific |
| 1003 | HDAC4 | ctDNA specific | 1034 | MED12 | ctDNA specific |
| 1003 | TP53 | ctDNA specific | 1034 | MLL2 | ctDNA specific |
| 1003 | ELAC2 | ctDNA specific | 1034 | ROS1 | ctDNA specific |
| 1003 | RARA | ctDNA specific | 1034 | TP53 | ctDNA specific |
| 1003 | NOTCH3 | ctDNA specific | 1034 | SERPINB4 | ctDNA specific |
| 1003 | IRS2 | ctDNA specific | 1034 | CIC | ctDNA specific |
| 1003 | BRCA2 | ctDNA specific | 1034 | NOTCH4 | ctDNA specific |
| 1003 | RB1 | ctDNA specific | 1034 | MSH6 | ctDNA specific |
| 1003 | GRIN2A | ctDNA specific | 1034 | RET | ctDNA specific |
| 1003 | CTCF | ctDNA specific |  |  |  |

Table S4. Tumor specific mutations detected from ctDNA analysis which were compared with WES data from 12 matched baseline tumor samples.

| ID | Gene | type | ID | Gene | type |
| --- | --- | --- | --- | --- | --- |
| 1002 | APC | tumor specific | 1017 | PMS1 | tumor specific |
| 1002 | PDGFRB | tumor specific | 1017 | PMS1 | tumor specific |
| 1002 | TP53 | tumor specific | 1017 | CASP8 | tumor specific |
| 1002 | CDK12 | tumor specific | 1017 | ERBB4 | tumor specific |
| 1002 | RARA | tumor specific | 1017 | SETD2 | tumor specific |
| 1002 | ATRX | tumor specific | 1017 | PDGFRA | tumor specific |
| 1003 | DDR2 | tumor specific | 1017 | FAT1 | tumor specific |
| 1003 | ABL2 | tumor specific | 1017 | CTNNA1 | tumor specific |
| 1003 | ALK | tumor specific | 1017 | HGF | tumor specific |
| 1003 | ALK | tumor specific | 1017 | RET | tumor specific |
| 1003 | LRP1B | tumor specific | 1017 | HNF1A | tumor specific |
| 1003 | LRP1B | tumor specific | 1017 | POLE | tumor specific |
| 1003 | HDAC4 | tumor specific | 1017 | BRCA2 | tumor specific |
| 1003 | FLT4 | tumor specific | 1017 | NTRK3 | tumor specific |
| 1003 | HGF | tumor specific | 1017 | TP53 | tumor specific |
| 1003 | NOTCH1 | tumor specific | 1017 | RAD51D | tumor specific |
| 1003 | BRCA2 | tumor specific | 1017 | AXIN2 | tumor specific |
| 1003 | IRS2 | tumor specific | 1017 | KDM6A | tumor specific |
| 1003 | GRIN2A | tumor specific | 1017 | MED12 | tumor specific |
| 1003 | CTCF | tumor specific | 1017 | MED12 | tumor specific |
| 1003 | TP53 | tumor specific | 1017 | MED12 | tumor specific |
| 1003 | TP53 | tumor specific | 1017 | MED12 | tumor specific |
| 1003 | ELAC2 | tumor specific | 1019 | EPHB2 | tumor specific |
| 1003 | RARA | tumor specific | 1019 | CDC73 | tumor specific |
| 1003 | NOTCH3 | tumor specific | 1019 | NOTCH1 | tumor specific |
| 1003 | GNAS | tumor specific | 1019 | POLE | tumor specific |
| 1004 | DNMT3A | tumor specific | 1019 | BRCA2 | tumor specific |
| 1004 | LRP1B | tumor specific | 1019 | TP53 | tumor specific |
| 1004 | SETD2 | tumor specific | 1019 | NOTCH3 | tumor specific |
| 1004 | ATR | tumor specific | 1027 | NTRK1 | tumor specific |
| 1004 | DAXX | tumor specific | 1027 | SMO | tumor specific |
| 1004 | RB1 | tumor specific | 1027 | MLH3 | tumor specific |
| 1004 | IRS2 | tumor specific | 1027 | TP53 | tumor specific |
| 1004 | HSP90AA1 | tumor specific | 1027 | SMAD4 | tumor specific |
| 1004 | ZFHX3 | tumor specific | 1030 | DDR2 | tumor specific |
| 1004 | TP53 | tumor specific | 1030 | LRP1B | tumor specific |
| 1004 | TP53 | tumor specific | 1030 | LRP1B | tumor specific |
| 1005 | MTOR | tumor specific | 1030 | ZFHX3 | tumor specific |
| 1005 | ERBB4 | tumor specific | 1030 | TP53 | tumor specific |
| 1005 | KIT | tumor specific | 1030 | MED12 | tumor specific |
| 1005 | FAT1 | tumor specific | 1030 | MED12 | tumor specific |
| 1005 | FAT2 | tumor specific | 1031 | CASP8 | tumor specific |
| 1005 | FGFR4 | tumor specific | 1031 | EGFR | tumor specific |
| 1005 | ESR1 | tumor specific | 1031 | GATA3 | tumor specific |
| 1005 | POLE | tumor specific | 1031 | CYLD | tumor specific |
| 1005 | SLX4 | tumor specific | 1031 | TP53 | tumor specific |
| 1005 | TP53 | tumor specific | 1034 | TP73 | tumor specific |
| 1005 | TOP2A | tumor specific | 1034 | PTCH2 | tumor specific |
| 1005 | BCOR | tumor specific | 1034 | NOTCH2 | tumor specific |
| 1005 | AR | tumor specific | 1034 | NTRK1 | tumor specific |
| 1008 | LRP1B | tumor specific | 1034 | MSH6 | tumor specific |
| 1008 | MSH3 | tumor specific | 1034 | ERBB4 | tumor specific |
| 1008 | ARID1B | tumor specific | 1034 | ATR | tumor specific |
| 1008 | RB1 | tumor specific | 1034 | KDR | tumor specific |
| 1008 | TP53 | tumor specific | 1034 | NOTCH4 | tumor specific |
| 1008 | EP300 | tumor specific | 1034 | ARID1B | tumor specific |
| 1015 | ALK | tumor specific | 1034 | RET | tumor specific |
| 1015 | LRP1B | tumor specific | 1034 | C11orf30 | tumor specific |
| 1015 | EPHA5 | tumor specific | 1034 | ATM | tumor specific |
| 1015 | FLT1 | tumor specific | 1034 | BRCA2 | tumor specific |
| 1015 | TP53 | tumor specific | 1034 | TP53 | tumor specific |
| 1015 | STAG2 | tumor specific | 1034 | SERPINB4 | tumor specific |
| 1015 | PHF6 | tumor specific | 1034 | CIC | tumor specific |

Table S5. Cell death genesets.

| Intrinsic_apoptosis | ABL1,ACKR3,AEN,AIFM1,AKT1,APAF1,ARHGEF2,ARL6IP5,ARMC10,ATAD5,ATF2,ATF4,ATM,ATP2A1,ATP2A3,BAD,BAG5,BAG6,BAK1,BAX,BBC3,BCAP31,BCL2,BCL2A1,BCL2L1,BCL2L10,BCL2L11,BCL2L2,BCL3,BCLAF1,BDKRB2,BECN1,BID,BNIP3,BOK,BRCA1,BRCA2,BRSK2,CASP2,CASP3,CASP4,CASP6,CASP9,CAV1,CCAR2,CD24,CD44,CD74,CDIP1,CDKN1A,CDKN2D,CEBPB,CHAC1,CHEK2,CIDEB,CLU,COA8,CREB3,CREB3L1,CRIP1,CUL1,CUL2,CUL3,CUL4A,CUL5,CXCL12,CYLD,CYP1B1,DAB2IP,DAPK2,DDIAS,DDIT3,DDIT4,DDX3X,DDX5,DIABLO,DNAJA1,DNAJC10,DNM1L,DYRK2,E2F1,E2F2,EDA2R,EIF2AK3,EIF5A,ELL3,ENO1,EP300,EPHA2,EPO,ERCC6,ERN1,ERN2,ERO1A,ERP29,FBH1,FBXW7,FHIT,FIGNL1,FIS1,FLCN,FNIP2,GPX1,GRINA,GSDME,GSKIP,HDAC1,HELLS,HERPUD1,HIC1,HIF1A,HINT1,HIPK1,HIPK2,HMOX1,HNRNPK,HRAS,HSPA1A,HSPB1,HTRA2,HYOU1,IFI16,IFI6,IKBKE,IL19,IL20RA,ING2,INS,ITPR1,IVNS1ABP,JAK2,JMY,KDM1A,LCK,LGALS12,LRRK2,MAEL,MAGEA3,MAP3K5,MAPK7,MAPK8IP1,MARCHF7,MCL1,MDM2,MELK,MIF,MIR132,MIR15A,MIR16-,MIR17,MIR186,MIR21,MIR27B,MLH1,MLLT11,MMP9,MOAP1,MSH6,MSX1,MUC1,MYBBP1A,MYC,NACC2,NBN,NCK1,NCK2,NDUFA13,NDUFS3,NFATC4,NFE2L2,NKX3,NME5,NOC2L,NOL3,NONO,NOX1,NUPR1,OPA1,P4HB,PARK7,PARL,PARP1,PDCD10,PDK1,PDK2,PDX1,PERP,PHLDA3,PIAS4,PIK3CB,PIK3R1,PINK1,PLAGL2,PLAUR,PLEKHF1,PMAIP1,PML,POLB,POU4F1,POU4F2,PPIA,PPIF,PPM1F,PPP1R13B,PPP1R15A,PRKCD,PRKDC,PRKN,PRKRA,PRODH,PTGS2,PTPMT1,PTPN1,PTPN2,PTTG1IP,PYCARD,QRICH1,RACK1,RAD9A,RIPK3,RNF183,RNF186,RPL26,RPS27L,RPS3,RPS7,RRM2B,RRN3,RRP8,RTKN2,S100A8,S100A9,SCN2A,SELENOK,SELENOS,SEPTIN4,SERINC3,SFN,SFPQ,SFRP2,SGMS1,SGPP1,SHISA5,SIAH1,SIRT1,SKIL,SLC9A3R1,SNAI1,SNAI2,SNW1,SOD1,SOD2,SRC,STK11,STK24,STK25,STYXL1,SYVN1,TAF9,TAF9B,TMBIM6,TMEM109,TMEM117,TMEM161A,TNF,TNFRSF10B,TNFRSF1A,TNFRSF1B,TOPORS,TP53,TP53BP2,TP63,TP73,TPT1,TRAF2,TRAP1,TREM2,TRIAP1,TRIB3,TRIM32,TXNDC12,UBB,UBQLN1,URI1,USP28,USP47,VDAC2,VNN1,WFS1,WWOX,XBP1,YBX3,ZNF385A,ZNF385B,ZNF622 |
| --- | --- |
| Extrinsic_apoptosis | ACVR1,ACVR1B,AGT,AGTR2,AKT1,AR,ARHGEF2,ATF3,BAD,BAG3,BAK1,BAX,BCL10,BCL2,BCL2A1,BCL2L1,BCL2L10,BCL2L11,BCL2L12,BCL2L14,BCL2L2,BEX3,BID,BIRC6,BLOC1S2,BMP4,BMP5,BMPR1B,BOK,BRCA1,CASP2,CASP8,CASP8AP2,CAV1,CD27,CD70,CFLAR,CIB1,COL2A1,CRADD,CSF2,CTNNA1,CTTN,CX3CL1,CYLD,DAB2IP,DAPK1,DAXX,DBH,DDX3X,DDX47,DEDD,DEDD2,DELE1,DEPTOR,DIABLO,ERBB3,EYA1,EYA2,EYA3,EYA4,FADD,FAF1,FAIM,FAIM2,FAS,FASLG,FEM1B,FGA,FGB,FGF10,FGFR1,FGFR3,FGG,FOXO3,G0S2,GABARAP,GATA1,GCLC,GCLM,GDNF,GFRAL,GPER1,GPX1,GSK3A,GSK3B,GSTP1,HGF,HIPK1,HMGB2,HMOX1,HSPA1A,HSPA1B,HTRA2,HTT,HYAL2,ICAM1,IFI27,IFI6,IFNG,IGF1,IL12A,IL19,IL1A,IL1B,IL2,IL4,IL6R,IL7,INHBA,ITGA6,ITGAV,ITM2C,ITPRIP,JAK2,KITLG,KRT18,LGALS3,LMNA,LTBR,MADD,MAL,MAP2K5,MAPK7,MCL1,MIR198,MIR221,MIR222,MKNK2,MLLT11,MOAP1,NDUFA13,NF1,NGF,NOL3,NOS3,NRP1,P2RX7,PAK2,PAK5,PARK7,PARP2,PDIA3,PDPK1,PEA15,PELI3,PF4,PHIP,PIDD1,PIK3R1,PMAIP1,PML,PPP1CA,PPP2R1A,PPP2R1B,PRDX2,PSME3,PTEN,PTPRC,PYCARD,RAF1,RB1CC1,RBCK1,RELA,RET,RFFL,RIPK1,RNF34,RNF41,RPS6KB1,SCG2,SCRT2,SERPINE1,SFRP1,SFRP2,SGPP1,SH3RF1,SIAH2,SIVA1,SKIL,SMAD3,SNAI2,SORT1,SP100,SPI1,SRC,SRPX,STK3,STK4,STRADB,STX4,TCF7L2,TERT,TGFB1,TGFB2,TGFBR1,THBS1,TIMP3,TLR3,TMBIM1,TMC8,TNF,TNFAIP3,TNFRSF10A,TNFRSF10B,TNFRSF10C,TNFRSF12A,TNFRSF1A,TNFRSF1B,TNFSF10,TNFSF12,TRADD,TRAF1,TRAF2,UNC5B,WWOX,YAP1,ZC3HC1,ZDHHC3,ZMYND11,ZSWIM2 |
| Pyroptosis | BAK1,BAX,CASP1,CASP3,CASP4,CASP5,CASP6,CASP8,CASP9,CHMP2A,CHMP2B,CHMP3,CHMP4A,CHMP4B,CHMP4C,CHMP6,CHMP7,CYCS,ELANE,GPX4,GSDMB,GSDMC,GSDMD,GSDME,GZMB,HMGB1,IL18,IL1A,IL1B,IRF1,IRF2,NLRC4,NLRP1,NLRP2,NLRP3,NLRP6,NLRP7,NOD1,PLCG1,PJVK,PRKACA,PYCARD,SCAF11,TINAP,TNF,TP53,TP63,AIM2,GSDMA,IL6,NOD2,TIRAP |
| Ferroptosis | ABCC1,ACACA,ACO1,ACSF2,ACSL1,ACSL3,ACSL4,ACSL5,ACSL6,AIFM2,AKR1C1,AKR1C2,AKR1C3,ALOX12,ALOX15,ALOX5,ATG5,ATG7,ATP5MC3,BACH1,CARS,CBS,CD44,CHAC1,CISD1,CP,CRYAB,CS,CYBB,DPP4,EMC2,FADS2,FANCD2,FDFT1,FTH1,FTL,FTMT,G6PD,GCLC,GCLM,GLS2,GOT1,GPX4,GSS,HMGCR,HMOX1,HSBP1,HSPB1,IREB2,KEAP1,LPCAT3,MAP1LC3A,MAP1LC3B,MAP1LC3C,MT1G,NCOA4,NFE2L2,NFS1,NOX1,NQO1,NRF2,OTUB1,PCBP1,PCBP2,PEBP1,PGD,PHKG2,PRNP,PROM2,PTGS2,RPL8,SAT1,SAT2,SLC11A2,SLC1A5,SLC39A14,SLC39A8,SLC3A2,SLC40A1,SLC7A11,SQLE,STEAP3,TF,TFRC,TP53,VDAC2,VDAC3,ZEB1 |
| Autophagy | ABL1,ABL2,ACER2,ADRA1A,ADRB2,AKT1,AMBRA1,ATF6,ATG101,ATG13,ATG14,ATG2A,ATG2B,ATG5,ATG7,ATM,ATP13A2,ATP6V0A1,ATP6V0A2,ATP6V0B,ATP6V0C,ATP6V0D1,ATP6V0D2,ATP6V0E1,ATP6V0E2,ATP6V1A,ATP6V1B1,ATP6V1B2,ATP6V1C1,ATP6V1C2,ATP6V1D,ATP6V1E1,ATP6V1E2,ATP6V1G1,ATP6V1G2,ATP6V1H,AUP1,BAD,BAG3,BCL2,BCL2L11,BECN1,BMF,BNIP3,BNIP3L,BOK,C9orf72,CALCOCO2,CAMKK2,CAPN1,CAPNS1,CASP1,CASP3,CDC37,CDK5,CDK5R1,CHMP4A,CHMP4B,CISD2,CLEC16A,CLN3,CLU,CPTP,CSNK2A2,CTSA,CTTN,DAP,DAPK1,DAPK2,DAPK3,DAPL1,DCN,DDIT3,DDRGK1,DEPDC5,DEPP1,DHRSX,DNM1L,DRAM1,DRAM2,EEF1A1,EEF1A2,EIF2AK4,EIF4G1,EIF4G2,ELAPOR1,EP300,EPM2A,ERCC4,ERN1,EXOC1,EXOC4,EXOC7,EXOC8,FBXL2,FBXO7,FBXW7,FEZ1,FEZ2,FLCN,FOXK1,FOXK2,FOXO1,FOXO3,FTH1,FTL,FYCO1,FZD5,GAPDH,GATA4,GBA,GFAP,GNAI3,GOLGA2,GPR137,GPR137B,GPSM1,GSK3A,GSK3B,HAX1,HDAC6,HERC1,HGF,HIF1A,HMGB1,HMOX1,HSP90AA1,HSPA8,HSPB1,HSPB8,HTR2B,HTRA2,HTT,HUWE1,IFI16,IFNG,IKBKG,IL10,IL10RA,IL4,IRGM,ITPR1,KAT5,KAT8,KDM4A,KDR,KEAP1,KIF25,KLHL22,KLHL3,LACRT,LAMP1,LAMP2,LAMP3,LAMTOR1,LAMTOR2,LAMTOR3,LAMTOR4,LAMTOR5,LARP1,LEP,LEPR,LGALS8,LRRK2,LRSAM1,LZTS1,MAP1LC3A,MAP1LC3B,MAP1LC3C,MAP3K7,MAPK15,MAPK3,MAPK8,MAPT,MCL1,MEFV,MET,MFN2,MFSD8,MID2,MIR199A1,MIRLET7B,MLST8,MT3,MTCL1,MTDH,MTM1,MTMR3,MTMR4,MTMR8,MTMR9,MTOR,NCOA4,NEDD4,NLRP6,NOD1,NOD2,NPC1,NPRL2,NRBP2,NUPR1,OPTN,ORMDL3,OSBPL7,PAFAH1B2,PARK7,PHB2,PHF23,PIK3C2A,PIK3C3,PIK3CA,PIK3CB,PIK3R2,PIM2,PINK1,PIP4K2A,PIP4K2B,PIP4K2C,PJVK,PLEKHF1,PLK2,PLK3,POLDIP2,PRKAA1,PRKAA2,PRKAB1,PRKAB2,PRKACA,PRKAG1,PRKAG2,PRKAG3,PRKD1,PRKN,PSAP,PTPN22,PYCARD,QSOX1,RAB39B,RAB3GAP1,RAB3GAP2,RAB7A,RAB8A,RALB,RASIP1,RB1CC1,RETREG1,RETREG3,RHEB,RIPK2,RMC1,RNF152,RNF41,RNF5,ROCK1,RPTOR,RRAGA,RRAGB,RRAGC,RRAGD,RUBCN,RUFY4,SCFD1,SCOC,SEC22B,SESN1,SESN2,SESN3,SH3BP4,SH3GLB1,SIRT1,SIRT2,SLC38A9,SMCR8,SMG1,SNCA,SNRNP70,SNX32,SNX5,SNX6,SOGA1,SOGA3,SPTLC1,SPTLC2,SQSTM1,SREBF1,SREBF2,STAT3,STBD1,STING1,STK11,STUB1,SUPT5H,SVIP,SYNPO2,TAB2,TAB3,TBC1D14,TBC1D25,TBK1,TEX264,TFEB,TICAM1,TIGAR,TLK2,TMEM150A,TMEM150B,TMEM150C,TMEM39A,TMEM39B,TMEM59,TOMM7,TP53,TP53INP1,TP53INP2,TPCN1,TPCN2,TREM2,TRIB3,TRIM13,TRIM14,TRIM21,TRIM22,TRIM27,TRIM34,TRIM38,TRIM5,TRIM6,TRIM65,TRIM68,TRIM8,TRIML1,TRIML2,TSC1,TSC2,TSPO,UBA5,UBQLN1,UBQLN2,UBQLN4,UCHL1,UFC1,UFL1,UFM1,ULK1,USP10,USP13,USP30,USP33,USP36,UVRAG,VDAC1,VPS13C,VPS13D,VPS26A,VPS26B,VPS35,WAC,WASHC1,WDFY3,WDR24,WDR41,WDR6,WDR81,WIPI2,ZC3H12A,ZKSCAN3,ZMPSTE24 |
| Necroptosis | GLUD1,GLUD2,ALOX15,FTH1,PYG,CAPN1,CASP1,GLNA,BAX,BCL2,FADD,RIPK1,TNF,TNFRSF1A,TRADD,TRAF2,PPIA,CAPN2,HSP90A,IL1A,TNFSF6,TNFRSF6,CASP8,JNK,JAK2,CAMK2,IL1B,IFNG,STAT3,IRF9,TNFSF10,TNFRSF10A,TNFRSF10B,CFLAR,XIAP,BID,AIFM1,TRPM7,IFNAR1,IFNAR2,IFNGR1,IFNGR2,TLR3,TIRP,IFNA,IFNB,TRIF,VDAC1,SLC25A4S,PPID,CYLD,RIPK3,MLKL,TRAF5,TLR4,RBCK1,HMGB1,JAK1,JAK3,TYK2,STAT1,STAT2,STAT4,STAT5A,STAT5B,STAT6,H2A,TNFAIP3,RNF31,CHMP2A,CHMP2B,VPS24,CHMP4A,CHMP4B,CHMP6,VPS4,CHMP1,CHMP5,SMPD1,PYCARD,NLRP3,ZBP1,IL33,FTL,SQSTM1,VDAC2,VDAC3,CHMP7,PGAM5,BIRC2,BIRC3,EIF2AK2,PLA2G4,DNM1L,SPATA2,FAF1,SHARPIN,NOX2,USP21,PARP1,CHMP4C |
| Cuproptosis | FDX1,LIAS,LIPT1,DLD,DLAT,PDHA1,PDHB,MTF1,GLS,CDKN2A,GCSH,ATP7A,ATP7B,SLC31A1 |
| Parthanatos | PARP,MIF,AIFM1,HSP70,PAAN,ARH3,RNF146,ADPRHL2,OGG1 |
| Entotic_cell_death | AMPK,ATG5,ATG7,BECN1,CDC42,CDH1,CTNNA1,CYBB,MYH14,PI3KC3,RHOA,RNF146,ROCK,RUBCN,UVRAG |
| Netotic_cell_death | ELANE,MMP1,MPO,CAMP,PADI4,EIPA,NCX1,MIA |
| Lysosome_dependent_cell_death | ABCA2,ABCB9,ACP2,ACP5,ADGRE2,AGA,AP1B1,AP1G1,AP1M1,AP1M2,AP1S1,AP1S2,AP1S3,AP3B1,AP3B2,AP3D1,AP3M1,AP3M2,AP3S1,AP3S2,AP4B1,AP4E1,AP4M1,AP4S1,ARF1,ARL8B,ARSA,ARSB,ARSG,ASAH1,ATP10B,ATP13A2,ATP6AP1,ATP6V0A1,ATP6V0A2,ATP6V0A4,ATP6V0B,ATP6V0C,ATP6V0D1,ATP6V0D2,ATP6V1H,BLK,BLOC1S1,BLOC1S2,BORCS5,BORCS6,BTK,C12orf4,CBL,CD164,CD300A,CD63,CD68,CD84,CHGA,CLN3,CLN5,CLNK,CLTA,CLTB,CLTC,CLTCL1,CLU,CPLX2,CTNS,CTSA,CTSB,CTSC,CTSD,CTSE,CTSF,CTSG,CTSH,CTSK,CTSL,CTSO,CTSS,CTSV,CTSW,CTSZ,DEF8,DNASE2,DNASE2B,ENTPD4,FAM98A,FER,FES,FGR,FLCN,FOXF1,FTH1,FTL,FUCA1,GAA,GAB2,GALC,GALNS,GATA2,GBA,GCC2,GGA1,GGA2,GGA3,GLA,GLB1,GM2A,GNPTAB,GNPTG,GNS,GUSB,HDAC6,HEXA,HEXB,HGS,HGSNAT,HMOX1,HPS6,HSPA8,HYAL1,IDS,IDUA,IGF2R,IL13,IL13RA2,IL4,IL4R,KIF1B,KIT,KXD1,LAMP1,LAMP2,LAMP3,LAMTOR1,LAPTM4A,LAPTM4B,LAPTM5,LAT,LAT2,LGALS9,LGMN,LIPA,LRRK2,LYN,M6PR,MAN2B1,MANBA,MAP1LC3A,MAP6,MCOLN1,MFSD8,MILR1,MRGPRX2,MT3,MYH9,NAGA,NAGLU,NAGPA,NAPSA,NCOA4,NDEL1,NEDD4,NEU1,NPC1,NPC2,NR4A3,PDPK1,PIK3C3,PIK3CD,PIK3CG,PIP4K2A,PIP4K2B,PIP4P1,PLA2G15,PLA2G3,PLEKHM1,PLEKHM2,PPT1,PPT2,PSAP,PSAPL1,PTGDR,PTGDS,RAB34,RAB3A,RAB7A,RAC2,RUBCNL,S100A13,SCARB2,SGSH,SLC11A1,SLC11A2,SLC17A5,SMPD1,SNAP23,SNAPIN,SNX16,SNX4,SORL1,SORT1,SPAG9,SPHK2,SQSTM1,STXBP1,STXBP2,SUMF1,SYK,SYTL4,TCIRG1,TFEB,TMEM106B,TPP1,UNC13D,VAMP7,VAMP8,VPS33A,VPS33B,VPS4A,WASH3P,ZFYVE16 |
| Alkaliptosis | IKBKB,NFKB1,CA9,CHUK,IKBKG,NFKB1A,RELA |
| Oxeiptosis | PGAM5,KEAP1,AIFM1,NRF2,AIRE |
| ICD | ATG5,BAX,CALR,CASP1,CASP8,CD4,CD8A,CD8B,CXCR3,EIF2AK3,ENTPD1,FOXP3,HMGB1,HSP90AA1,IFNA1,IFNB1,IFNG,IFNGR1,IL10,IL17A,IL17RA,IL1B,IL1R1,IL6,LY96,MYD88,NLRP3,NT5E,P2RX7,PDIA3,PIK3CA,PRF1,TLR4,TNF |
